# The acyltransferase PMAT1 malonylates brassinolide glucoside

**DOI:** 10.1016/j.jbc.2021.100424

**Published:** 2021-02-16

**Authors:** Sufu Gan, Wilfried Rozhon, Elisabeth Varga, Jyotirmoy Halder, Franz Berthiller, Brigitte Poppenberger

**Affiliations:** 1 Biotechnology of Horticultural Crops, TUM School of Life Sciences Weihenstephan, Technische Universität München, Freising, Germany; 2 Institute of Bioanalytics and Agro-Metabolomics, Department of Agrobiotechnology (IFA-Tulln), University of Natural Resources and Life Sciences, Vienna, Tulln, Austria

**Keywords:** brassinosteroids, catabolic inactivation, hormone, malonylation, steroid, ACC, aminocyclopropane-1-carboxylic acid, BL, brassinolide, BR, brassinosteroid, BR-Glc, BR glucoside, BR-MalGlc, BR malonylglucosides, BL-23-O-Glc, BL-23-O-glucoside, CoA, coenzyme A, CS, castasterone, malonylTFs, malonyltransferases, NASC, Nottingham Arabidopsis Stock Center, ORF, open reading frame, PMAT1, phenolic glucoside malonyl-transferase 1, UGT, glycosyltransferase

## Abstract

Brassinosteroids (BRs) are steroid hormones of plants that coordinate fundamental growth and development processes. Their homeostasis is controlled by diverse means, including glucosylation of the bioactive BR brassinolide (BL), which is catalyzed by the UDP-glycosyltransferases (UGTs) UGT73C5 and UGT73C6 and occurs mainly at the C-23 position. Additional evidence had suggested that the resultant BL-23-*O*-glucoside (BL-23-*O*-Glc) can be malonylated, but the physiological significance of and enzyme required for this reaction had remained unknown. Here, we show that in *Arabidopsis thaliana* malonylation of BL-23-*O*-Glc is catalyzed by the acyltransferase phenolic glucoside malonyl-transferase 1 (PMAT1), which is also known to malonylate phenolic glucosides and lipid amides. Loss of PMAT1 abolished BL-23-*O*-malonylglucoside formation and enriched BL-23-*O*-Glc, showing that the enzyme acts on the glucoside. An overexpression of PMAT1 in plants where *UGT73C6* was also overexpressed, and thus, BL-23-*O*-Glc formation was promoted, enhanced the symptoms of BR-deficiency of *UGT73C6oe* plants, providing evidence that PMAT1 contributes to BL inactivation. Based on these results, a model is proposed in which PMAT1 acts in the conversion of both endogenous and xenobiotic glucosides to adjust metabolic homeostasis in spatial and temporal modes.

The maintenance of steroid hormone homeostasis is of high relevance for animals and plants alike. The levels of steroids can be controlled by multiple means including catabolic inactivation, and glycosylation plays a key role. In humans, androgens and estrogens are conjugated by UDP-glycosyltransferases (UGTs) with glucuronic acid, which reduces bioavailability and promotes secretion (1, 2). These reactions are thought to be mostly irreversible, although a release of the hormonal aglycons through the activity of β-glucuronidases can occur, for example by β-glucuronidase activities of microbes in the gut (3).

In plants, homeostasis of the steroid hormones brassinosteroids (BRs) is also regulated by glycosylation (4). BRs are polyhydroxylated sterols formed from campesterol by several P450 monooxygenases, including CPD/CYP90A1, DWF4/CYP90B1, and ROT3/CYP90D1 (5). Castasterone (CS) and brassinolide (BL) are the biologically most active BRs, and both can be conjugated with glucose at the C-23 position, via activity of the UGTs UGT73C5 and UGT73C6. An overexpression of these UGTs decreases endogenous BR levels and induces characteristic BR-deficient phenotypes, including dwarfism, delayed flowering, and senescence, as well as reduced fertility (4, 6).

While there is evidence that glucose conjugation is an inactivation reaction, the physiological consequences of BR-glucoside formation are unclear. Also, it is unknown if BR-glucosides can be reactivated, although there are indications that they can undergo further conjugation with acyl groups, the relevance of which is not known (6). Acylation is a common catabolic modification used for decoration of hydroxy group-containing small molecular weight compounds and is catalyzed by acyltransferases (7). Aliphatic acylation can promote the compartmentalization of glycosides and/or inhibit degradation by β-glucuronidases and β-glucosidase, thereby preventing reactivation of the aglycons (8, 9). In plants, common aliphatic and aromatic acyl donors are coenzyme A (CoA) thioesters, which are utilized by BAHD-type acyltransferases to modify secondary metabolites with diverse functions, including the pigments anthocyanins, volatile esters, and preformed defense compounds (8, 9, 10). But also BRs can be acetylated. The BAHD acyltransferase BIA1 (BR INACTIVATOR 1) uses acetyl-CoA as a donor for monoacetylation and diacetylation of CS (11), and PIZZA/BAT1 can modify BRs by conjugation with long-chain fatty acids (12). When malonic acid is attached to glucose moieties of acceptors malonyl glucosides are formed (8, 9, 10), and first results suggested that BR glucoside (BR-Glc) can be converted to BR malonylglucosides (BR-MalGlc) in *Arabidopsis thaliana*, which appeared to be more stable catabolites (6).

Here, we show that malonylation of BL-23-*O*-Glc is catalyzed by the BAHD acyltransferase PMAT1 (phenolic glucoside malonyl-transferase 1), which can also malonylate phenolic glucosides and lipid amides (9, 10). PMAT1 and its homolog At5MAT accepted BL-23-*O*-Glc as substrate *in vitro* and were positively regulated by BR signaling. In *PMAT1* knock-out plants, BL-23-*O*-MalGlc formation was abolished, and BL-23-*O*-Glc levels were elevated, showing that PMAT1 is required for the malonylation of BL-Glc and that this activity promotes BL-Glc turn-over. Overexpression of *PMAT1* in a background in which the UDP-glucosyltransferase *UGT73C6* was also overexpressed, and thus BR-glucoside levels were elevated, increased BL-MalGlc amounts, and enhanced symptoms of BR-deficiency in *UGT73C6oe* plants, providing evidence that malonylation of BL-Glc decreased BL bioavailability. A model for PMAT1 activity in BR homeostasis is presented, and its dual roles in hormone catabolism and detoxification of xenobiotics is discussed.

## Results

### PMAT1 and At5MAT catalyze formation of BR-MalGlc *in vitro*

In plants overexpressing the UGTs *UGT73C5* or *UGT73C6*, the formed BL-23-*O*-Glc is rapidly converted to BL-23-*O*-MalGlc (6). To isolate enzymes involved in BR-23-*O*-Glc malonylation, a candidate gene approach was taken. BLASTP searches against the Araport11 protein sequence database were conducted with PMAT1 (encoded by locus *At5g39050*), for which there was evidence for *in vivo* malonylation activity (9, 10). This identified 55 putative *Arabidopsis* BAHD acyltransferases that contained the conserved BAHD signature motifs HxxxD and DFGWG, which was consistent with previous reports (13, 14). A phylogenetic tree was assembled and showed that the enzymes sorted into four major clades (Fig. S1*A*), again being consistent with previous results (13, 14). Eight enzymes of a sub-branch of clade II (highlighted with a gray bar in Fig. S1*A*) were selected as the most promising candidates for BR-Glc malonylation, because PMAT1, PMAT2, and At5MAT, who are members of this subclade, had previously been shown to use malonyl-CoA as a donor substrate (9, 10, 15). An amino acid alignment of BAHD signature domain-containing areas showed a high conservation between the eight proteins (Fig. S1*B*).

**Figure 1:**
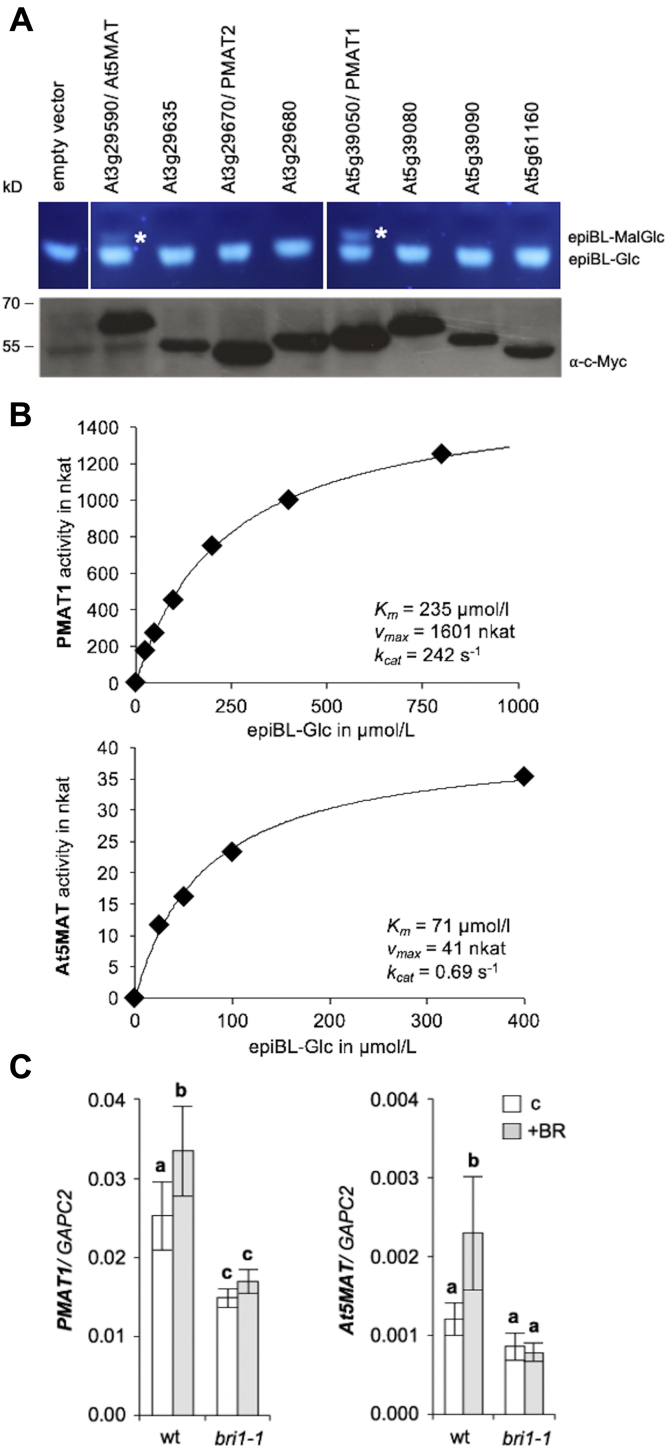
**PMAT1 and At5MAT catalyze epiBL-Glc malonylation *in vitro* and are induced by BR signaling.***A*, activity of *in vitro-*translated clade II BAHD acyltransferases against epiBL-23-*O*-Glc. *Upper panel*: *In vitro* malonylation reactions were carried out with 5 μl of *in vitro-*translated enzyme preparation, epiBL-23-*O*-Glc as an acceptor and malonyl-CoA as a donor substrate. The reaction products were separated by TLC and visualized with UV light (at 366 nm). The position of the reaction products is indicated with asterisks. *Lower panel*: Immunoblot analysis of the *in vitro-*translated enzymes using an anti-c-Myc antibody. Five μL of *in vitro-*translated samples were loaded. *B,* Enzyme kinetics of GST-tagged PMAT1 and At5MAT using epiBL-Glc as substrate. Recombinant proteins were used for malonylation assays in 50 mM sodium phosphate buffer pH 8.0, and epiBL-Glc at the indicated concentrations. The products were analyzed by TLC. *C,* Expression of *PMAT1* and *At5MAT* in wild-type (wt) and *bri1-1* following BR-treatment. Plants were grown on ½ MS plates for 10 days and then transferred to liquid ½ MS medium supplemented with 1 μM 24-epiBL for 24 h (+BR). ½ MS medium supplemented with dimethyl sulfoxide was used as a control (c). qPCRs were carried out with gene-specific primers and *GAPC2* for normalization. Values are the means and standard deviations (SDs) of four biological replicates measured in four technical repeats. Letters indicate significant differences (*p* < 0.05; one-way ANOVA, Tukey post-hoc test). BR, brassinosteroid; CoA, coenzyme A; PMAT1, phenolic glucoside malonyl-transferase 1.

To test if clade II members can catalyze BR-MalGlc formation, enzymatic activity assays with the putative malonyltransferases (malonylTFs) were carried out. A wheat germ extract–based protein translation system was used for the production of c-Myc–tagged proteins and worked efficiently (Fig. 1*A*). In addition, the acceptor substrate had to be generated, because it is not commercially available. For this purpose, *in vitro* glucosylation assays, with recombinant GST-tagged UGT73C5, UDP-glucose as a donor, and 24-epiBL (epiBL) as an acceptor were carried out, and the resulting epiBL-23-*O*-Glc was purified by preparative HPLC. The malonylTFs were then incubated with malonyl-CoA and epiBL-23-*O*-Glc, the reaction products were separated by thin layer chromatography (TLC) and visualized with UV light at 366 nm wavelength. The results showed that an additional band was obtained in the reactions with PMAT1 and also with At5MAT (encoded by locus *At3g29590*), albeit at a lower level for the latter (Fig. 1*A*). No products were seen with any of the other enzymes in this system, and thus, PMAT1 and At5MAT were selected for further characterization.

**Figure 2:**
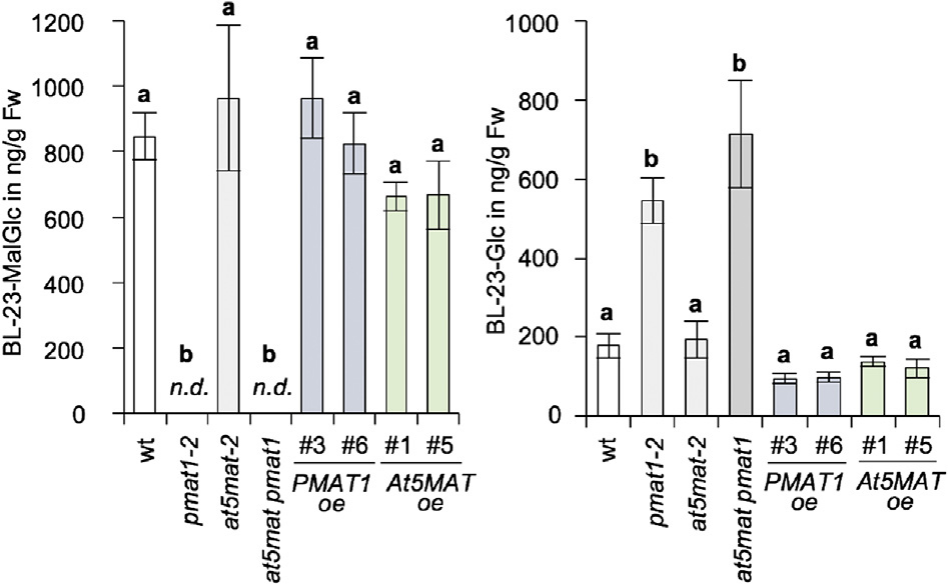
**PMAT1 is required for malonylation of BL-23-*O*-Glc *in planta*.** Levels of BL-23-*O*-MalGlc (*left*) and BL-23-*O*-Glc (*right*) in ng/g fresh weight (Fw) in BL-treated plants of the indicated lines. Eleven-day-old seedlings were incubated with 1 μg/ml BL for 48 h, and following extraction, the samples were analyzed by HPLC-QTOF. Values are the means and SD of 3 to 4 replicates; n.d., not detected. The letters indicate significantly different values (*p* < 0.05; one-way ANOVA, Tukey post-hoc test). BL, brassinolide; BL-23-*O*-Glc, BL-23-*O*-glucoside; BL-23-*O*- MalGlc, BL-23-*O*-malonylglucosides; PMAT1, phenolic glucoside malonyl-transferase 1.

For verification of the catalytic activities, PMAT1 and At5MAT were expressed as GST-fusion proteins in *Escherichia coli* BL21, the activity assays with epiBL-23-*O*-Glc and malonyl-CoA were repeated, and different pH buffer conditions and input protein amounts were tested. Both enzymes preferred neutral to slight alkaline conditions, with best conversion rates at pH 7.0 to pH 8.5 (Fig. S2*A*). To compare the catalytic activity of the recombinant enzymes, reactions with different input protein amounts were performed and analyzed by TLC (Fig. S2*B*). Quantification of the product indicated that PMAT1 has a much higher activity than At5MAT for malonylation of epiBL-Glc. To investigate this in more detail, an enzyme kinetic study was performed, which showed that At5MAT had a higher affinity for BL-Glc than PMAT1 *in vitro*, as shown by the *K*_*m*_ values of 71 μmol/l and 235 μmol/l, respectively (Fig. 2*B*). In contrast, PMAT1 had a much higher turnover rate (*k*_*cat*_) than At5MAT (Fig. 1*B*) and a specificity constant (*k_cat_*/*K_m_*) of 1.0 × 10^6^ L × mol^-1^ × s^-1^, which is approximately 100 times higher than that of At5MATs with 9.7 × 10^3^ L × mol^-1^ × s^-1^ (Table S1). In this respect, it is important to note that *k*_*cat*_/*K*_*m*_ values for malonylation of other substrates, for instance cyanidin, pelargonidin, and peonidin-3,5-diglucoside, by At5MAT are in the range of 10^6^ (Table S1; (8)), which indicated that BL-Glc is not a preferred substrate of At5MAT *in vitro*. The malonylation reactions of epiBL-Glc and kaempferol-7-*O*-glucoside catalyzed by PMAT1 have both similarly high specificity constants (Table S1) indicating that both compounds are preferably used. In contrast, glucosides of xenobiotic compounds including naphthol glucosides and 4-nitrophenyl glucoside are clearly less efficiently malonylated as indicated by the lower *k*_*cat*_/*K*_*m*_ values.

In summary, there is evidence that At5MAT and in particular PMAT1 can catalyze the transfer of a malonyl moiety from malonyl-CoA to epiBL-23-*O*-Glc *in vitro*.

### PMAT1 and At5MAT are positively regulated by BR signaling

Enzymes involved in catabolic inactivation of hormones are often induced by the hormones signaling cascades to feedback-adjust homeostasis, and also genes encoding BR-catabolizing enzymes, such as the cytochrome P450 *BAS1* and the BAHD acyltransferase *BIA1* are BR-induced (16, 17). To investigate, if *PMAT1* and *At5MAT* are BR responsive, qPCR analyses of epiBL-treated WT plants were performed. This showed that when whole seedlings were analyzed, both genes were found to be slightly, but significantly BL-induced. Moreover, in *bri1-1*, a null allele mutant of the BR receptor BRI1 that abolishes BR signaling (18), *PMAT1* expression was constitutively repressed, and the expression of both genes was not responsive to epiBL (Fig. 1*C*). Thus, BR signaling can promote *PMAT1* and *At5MAT* transcription.

### A loss of PMAT1 function abolishes BL-23-O-MalGlc formation

To explore a potential function of the two malonylTFs in BR catabolism *in planta*, T-DNA-insertion lines with predicted insertions in the open reading frames (ORFs) of the genes were ordered from the Nottingham Arabidopsis Stock Center (NASC) and sequenced. Line SALK_007564 is *pmat1-2* (10), and in agreement with the published work, the T-DNA was found to be integrated at position 538 (after the start codon) of the *PMAT1* ORF. Line SM_3_35,619 harbors a T-DNA in the *At5MAT* ORF at position 929. Since a first *at5mat* knock-out allele had already been described (15), this new allele was named *at5mat-2*. Double *pmat1-2 at5mat-2* mutants were generated by crossing, and homozygosity was verified by genotyping the F3 generation. Semiquantitative PCRs confirmed that in the single and double mutants, expression of *PMAT1* and/or *At5MAT* was defective (Fig. S3*A*).

In addition to isolating knock-out mutants, overexpression lines were created. WT Col-0 was transformed with untagged *35S:PMAT1* or *35S:At5MAT* constructs, homozygous lines from independent transgenics were selected, and transgene expression was determined by qPCRs. This showed that *35S:PMAT1* (*PMAT1oe*) lines 3, 6, and 8 and *35S:At5MAT* (*At5MAToe*) lines 1, 5 and 10 had the highest levels of transgene expression, with increases of approximately 130- to 390-fold in case of the former and 560- to 660-fold in case of the latter (Fig. S3*B*), and thus, these lines were chosen for a characterization. For phenotypic comparison, the knock-out and over-expression lines were grown under standard growth conditions to the adult stage, where they did not exhibit obvious morphological defects (Fig. S3*C*).

The generated lines were then used to test, if altering *PMAT1* or *At5MAT* mRNA abundance may impact the BL-23-*O*-Glc malonylation capacities of plants. For this purpose, feeding experiments were performed with BL, because this increases BL-Glc concentrations to detectable amounts (endogenous levels are below the detection limit (4, 6)). Eleven-day-old seedlings of the knock-outs, two overexpression lines each and WT were incubated with 1 μg/ml BL in ½ MS media for 48 h, and following methanol extraction, the samples were analyzed by HPLC-QTOF using BL-23-*O*-Glc and BL-23-*O*-MalGlc as analytical reference, which were generated *in vitro* with recombinant UGT73C5 and PMAT1 (see supplementary methods). The identities of the reference compounds were confirmed by HR-MS and HR-MS/MS measurements (Figs. S4–S7). The result showed that while in seedlings of the single *at5mat-2* mutant BL-23-*O*-MalGlc levels were comparable to WT, BL-23-*O*-MalGlc was undetectable in the *pmat1-2* single and also in the *pmat1 at5mat* double mutant (Fig. 2). This was correlated with increased amounts of the BL-23-*O*-Glc acceptor in the *pmat1-2* single and *pmat1 at5mat* double mutant, providing evidence that the decreased conversion to BL-23-*O*-MalGlc enriched BL-23-*O*-Glc in the plants. In seedlings of the *PMAT1* and *At5MAT* overexpression lines, no significant differences as compared with WT were seen (Fig. 2).

To investigate if a loss of PMAT1 function alters plant growth or BR responses, we assessed root and hypocotyl elongation in seedlings of the single and double knock-outs, as well as the *PMAT1oe* lines, on media without or with epiBL and both in the light (Fig. S8, *A–C*) and in the dark (Fig. S8*D*); however, no differences to WT became apparent in this experimental set-up.

### An overexpression of PMAT1 enhances BR deficiency in UGT73C6oe plants

While PMAT1 efficiently catalyzed malonylation of BR-Glc *in vitro*, and a loss of PMAT1 function abolished BL-23-*O*-MalGlc formation *in planta*, *PMAT1oe* plants did not show significantly increased BL-23-*O*-MalGlc levels, at least not in the eleven-day-old seedlings that were used for analyses. Several reasons may account for this fact, one being insufficient BL-23-*O*-Glc acceptor availability in these lines. To test this hypothesis, *35S:PMAT1oe#8* was crossed with the *UGT73C6* over-expressing line *35S:UGT73C6-YFP-30* (6). *UGT73C6oe* plants accumulate large amounts of BR-23-*O*-Glc, have decrease levels of bioactive BRs, and show clear dwarfism and other typical signs of BR deficiency (6). In addition, also *35S:At5MAToe#10* was crossed with *35S:UGT73C6-YFP-30,* and F3 progeny homozygous for the transgenes was selected.

Because all used overexpression constructs are driven by 35S-promoters, it was verified by qPCRs, if a co-suppression of the transgenes occurred. This showed that in seedlings, *UGT73C6* expression was not co-repressed, neither in line *PMAT1oe x UGT73C6oe* nor in line *At5MAToe x UGT73C6oe*, and also *At5MAT* expression was not affected. Only *PMAT1* co-repression occurred to some extent in the double overexpressor *PMAT1oe x UGT73C6oe* (Fig. S9*A*).

**Figure 3:**
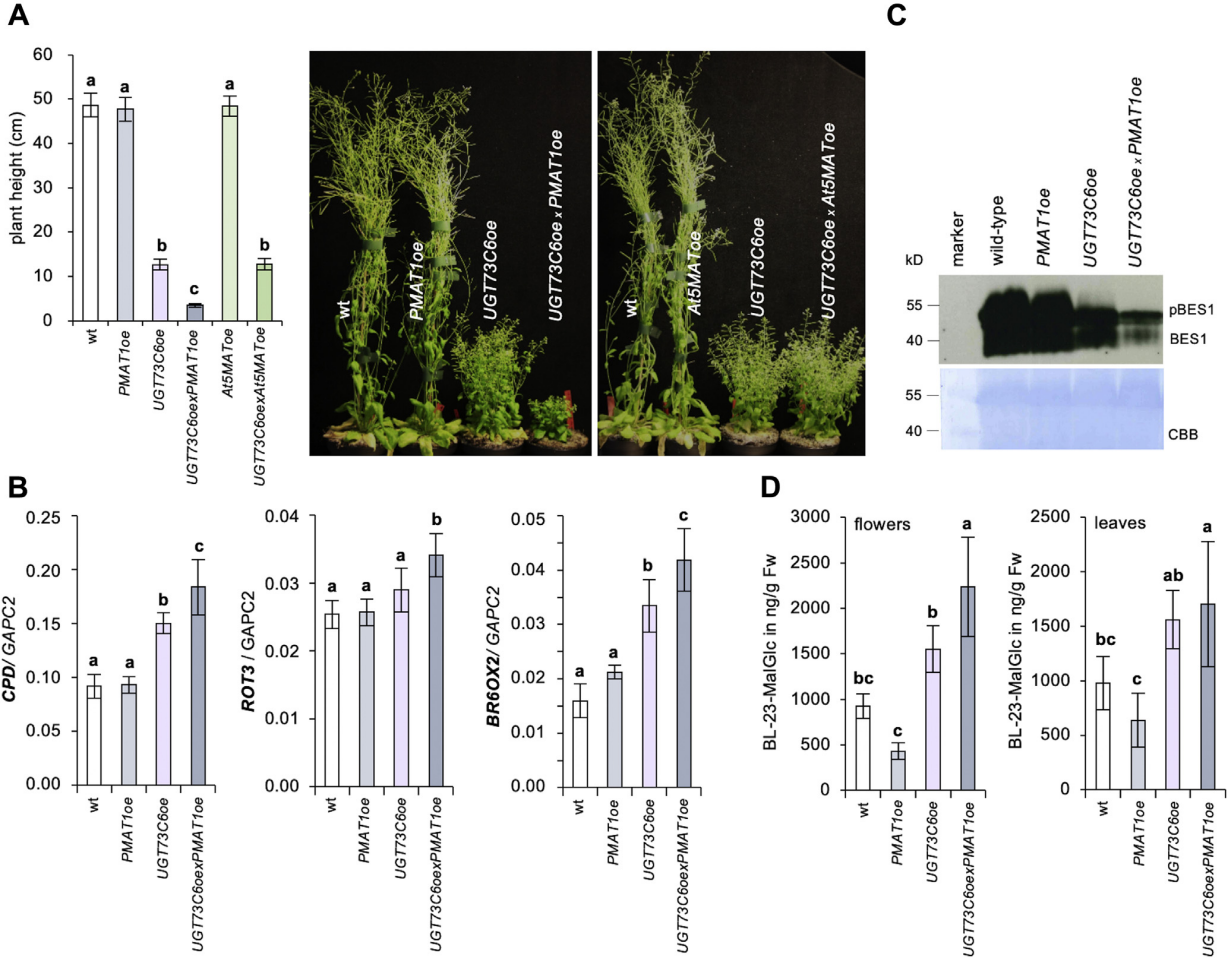
***PMAT1* over-expression enhances symptoms of BR-deficiency in *UGT73C6oe* plants.***A,* phenotypic evaluation of adult *UGT73C6oe x PMAT1oe* plants. *Left*: plant height of 8-week-old plants of *UGT73C6oe x PMAT1oe*, as compared with the parental lines and wildtype. The plants were grown in long days at 80 μmol m^−2^ s^−1^ of white light and 21 °C. Values are the means and SD of at least 12 plants. *Right*: photos of representative plants grown as on the *left* and compared to *At5MAToe* lines. *B,* expression of the BR-biosynthetic genes *CPD*, *ROT3,* and *BR6o x 2* in floral tissues of *UGT73C6oe x PMAT1oe,* the parental lines and wildtype, determined by qPCR. *GAPC2* was used for normalization. The means and SDs of 3 to 8 biological replicates measured in four technical repeats are shown. Letters show significant differences (*p* < 0.05; one-way ANOVA, Tukey post-hoc test). *C,* immunoblot analysis of BES1 with an ⍺-BES1 antibody from the same tissues as in B. Coomassie Brilliant Blue (CBB) staining was used for loading control. *D,* levels of BL-23-*O*-MalGlc in ng/g Fw in BL-treated plants of the indicated lines. Leaves and flowers of adult plants were treated with 1 μg/ml BL for 48 h, and the samples were analyzed by HPLC-QTOF. Values are the means and SDs of 3 to 4 replicates; n.d., not detected. Letters indicate significant differences (*p* < 0.05; one-way ANOVA, Tukey post-hoc test). BL-23-*O*-MalGlc, BL-23-*O*-malonylglucosides; BL, brassinolide; BR, brassinosteroid; PMAT1, phenolic glucoside malonyl-transferase 1; UGT, glycosyltransferase.

To investigate the phenotypic impact of introducing *35S:PMAT1* or *35S:At5MAT* into the *UGT73C6oe* background, several growth parameters were investigated in the F3 progeny of the crosses and compared with the parental lines and WT. Four weeks after germination, it was very apparent that the characteristic BR-deficient phenotype of the *UGT73C6oe* line was intensified in *PMAT1oe x UGT73C6oe*, because rosette size was significantly reduced. This effect was not seen in the *At5MAToe x UGT73C6oe* line (Fig. S9, *B* and *C*). Eight weeks after germination, the enhanced BR-deficient phenotype of *PMAT1oe x UGT73C6oe* became even more obvious: plant height and fertility were more strongly compromised, and senescence was further delayed as compared with the *UGT73C6oe* parent and again, *At5MAToe* did not produce these effects (Figs. 3*A*and S9*D*).

**Figure 4:**
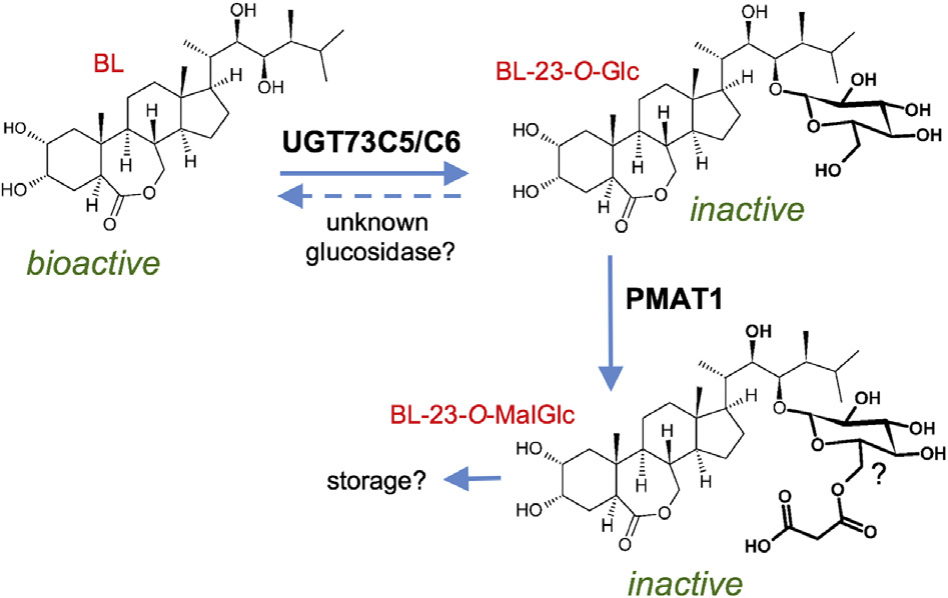
**Model for PMAT1 activity in BR homeostasis.** In *A. thaliana,* BL is converted to BL-23-*O*-Glc through activity of the UGTs UGT73C5 and UGT73C6. This inactive catabolite can be further catabolized to BL-23-*O*-MalGlc (by analogy to 2-naphthol-MalGlc (9) tentatively shown as a 6-*O’* malonylation product), which is a stabilizing reaction and requires PMAT1 function. Whereas the BL-23-*O*-Glc may be reactivated by unknown β-glucosidases to release bioactive BL, and malonylation in general is thought to be a modification that promotes compartmentalization for storage. BL, brassinolide; BL-23-*O*-Glc, BL-23-*O*-glucoside; BL-23-*O*-MalGlc, BL-23-*O*-malonylglucosides; BR, brassinosteroid; PMAT1, phenolic glucoside malonyl-transferase 1; UGT, glycosyltransferase.

To study if the enhanced BR-deficient phenotype of *PMAT1oe x UGT73C6oe* was because of decreased BL levels, the plants were sprayed with epiBL. In treated plants aspects of the phenotypes, such as the decreased rosette diameter where alleviated to some extent (Fig. S10), albeit the rescue was not complete, which is expected for plants with strongly increased BL-inactivation capacities. To further verify if BL activity was reduced in this line, two read-outs for BR signaling capacity were measured. On the one hand, the expression of the BR-biosynthetic genes *CPD*, *ROT3,* and *BR6ox2*, which are feedback-induced by BR deficiency (19, 20), was analyzed by qPCRs. On the other hand, the phosphorylation state of BES1 was determined by immunoblotting, using a BES1-specific antibody (21). BES1 is a transcription factor that is de-phosphorylated and stabilized by BRs (22, 23), and thus, in BR-deficient situations, an over-all reduction of BES1 levels and an enrichment of its phosphorylated forms occurs. The results showed that when compared with the parent lines and WT, the expression of all analyzed BR-biosynthetic genes was significantly elevated in the double overexpressor *PMAT1oe x UGT73C6oe* (Fig. 3*B*). This was correlated with an over-all reduction of BES1 (Fig. 3*C*), showing that BR signaling capacities were reduced. These decreased BR responses were associated with elevated BL-23-*O*-MalGlc formation (Fig. 3*D*), providing evidence that an increased capacity to malonylate BR-Glc promotes BR deficiency in plants.

## Discussion

Homeostasis of steroids must be controlled to allow for proper development, and catabolic inactivation by glycosylation plays a vital role in this process. In humans, steroid hormone glycosides can serve as storage forms, because the bioactive hormones can be reactivated by the action of β-glucuronidases (1, 2). This is of relevance for homeostatic regulation and, if miss-balanced, can result in disease development. For example, estrogen glycosides can be reactivated by microbial β-glucuronidase activities in the gut, which is thought to have an implication in breast cancer development (3).

In plants, the precise adjustment of BR homeostasis is of equal importance. A deficiency in BRs yields severe developmental defects including dwarf growth, delayed flowering, and decreased fertility (5). However, also an excess of bioactive BRs can be detrimental, with organ, developmental stage, and concentration-dependent effects. For example, an external application of BR, while promoting root elongation at low concentrations, represses root growth at elevated amounts (24). Thus, there is a clear requirement for a spatiotemporal control of BR homeostasis, and it is therefore perhaps not surprising that multiple modes have evolved, including a diverse set of catabolic inactivation events. In addition to hydroxylation, sulfonation and glucosylation (4, 25, 26) also acetylation can modify BRs. Recently, it has been shown that the BAHD acyltransferase BIA1 inactivates CS via monoacetylation and diacetylation, likely at the C-3 and/or C-22 or C-23 positions (11).

Here, we report that another BAHD acyltransferase, PMAT1, acts in the catabolism of BRs, albeit by a different means: through malonylation of BL-23-*O*-Glc (Fig. 4). PMAT1 catalyzed malonylation of epiBL-23-*O*-Glc *in vitro,* and in seedlings of a *pmat1* knock-out mutant, BL-23-*O*-MalGlc formation was lost, showing that PMAT1 is required for this activity. In seedling tissues, a loss of BL-23-*O*-MalGlc formation was also correlated with an increase in BL-23-*O*-Glc, showing that PMAT1 participates in the adjustment of BL-Glc levels. The fact that we did not see constitutive developmental defects or impaired growth responses to external BL in seedlings of these lines may have different not mutually exclusive reasons: (1) other modes of homeostatic regulation compensate for a loss of BL-Glc malonylation capacities in seedlings, (2) altered BR levels and/or responses prevail in specific cell types or developmental stages of these lines only and the phenotypic read-outs evaluated are not adequate to reveal defects, and/or (3) BL-MalGlc formation is not important for the adjustment of BR homeostasis.

A result that speaks against the last scenario is that clear symptoms of BR-deficiency developed when the production of endogenous BL-MalGlc levels was stimulated. To achieve a clear change at a whole plant level, an overexpression of *PMAT1* had to be paired with an overexpression of *UGT73C6*. *UGT73C6oe* increases BL-Glc abundance (6), and in this chemotypic background setting, *PMAT1oe* produced elevated BL-23-*O*-MalGlc concentrations and enhanced the symptoms of BR-deficiency of *UGT73C6oe* plants. This indicates that malonylation of BL-Glc is a reaction that further decreases the activity of BL for example by stabilization of the glucoside (Fig. 4). Such effects have been described for other malonylated products like anthocyanins. Anthocyanin-MalGlc show an increased stability at certain pHs and an enhanced resistance to β-glucosidase degradation as compared with the glucosides (27). Moreover, also for the plant hormone ethylene malonylation has been proposed to be recruited for homeostatic regulation. The ethylene precursor 1-aminocyclopropane-1-carboxylic acid (ACC) is malonylated by an as yet unknown ACC-*N*-malonylTF that utilizes malonyl-CoA to form the inactive catabolite malonyl-ACC, which is stored in the vacuole. This is thought to be of relevance during the ripening of climacteric fruits such as tomato and grapefruit, where ethylene stimulates malonylation of ACC for feedback adjustment of homeostasis (28).

In a situation where malonylation would impair a reactivation of BR-Glc by β-glucosidases, nonmalonylated BR-Glcs would serve as storage forms, and BR glucosylation would constitute a reversible inactivation reaction (Fig. 4), a mode that is also utilized by other plant hormones such as the auxin indole-3-acetic acid, the cytokinin zeatin, and abscisic acid for homeostatic regulation (29, 30, 31). However, to conclusively show the relevance of such means also for BR homeostasis will be challenging, because the bioactive BRs CS and BL are present in significantly lower amounts than indole-3-acetic acid, zeatin, or abscisic acid, hindering detection of the parent aglycon, let alone their glucosides or products thereof.

Another challenge in investigating the relevance and functional significance of BL-Glc and BL-MalGlc formation is redundancy with other modes of catabolism on the one hand, and functionally redundant enzymes on the other hand. In this context, an enzyme that may act redundantly with PMAT1 in the malonylation of BL-Glc in certain cell types is At5MAT, which showed *in vitro* activity against epiBL-23-*O*-Glc, albeit by a weaker extend then PMAT1. However, because a loss of At5MAT function did not impact BL-23-*O*-MalGlc formation abilities in seedlings and its overexpression in the *UGT73C6oe* background did not produce phenotypic changes in seedlings or adult plants, our results suggest that At5MAT does not contribute to BL-23-*O*-Glc malonylation in the developmental framework that we assessed.

In addition to the modification of plant secondary metabolites for increasing structural diversity, changed stability, and solubility, malonylation also provides a means of detoxification. It is part of the phase II detoxification system, where in consecutive reactions, reactive xenobiotics (potentially activated via hydroxylation in phase I) are first glycosylated, and the resulting glycosides are then further modified by malonylation, for deposition in dedicated cellular compartments such as the vacuole during phase III (32, 33). PMAT1 activity is required for the detoxification of the xenobiotic phenols 1-naphthol and 2-naphthol, via malonylation of the corresponding naphthol glucosides (9) and also for the malonylation of the lipid amides *N*-acylethanolamines, endogenous signaling molecules with unclear functions in plants (10). Because PMAT1 in addition malonylates BR glucosides, it is clear that it has dual roles in xenobiotic detoxification and endogenous signaling compound conversion *in planta*, accepting substrates with diverse structural features. Such a promiscuous activity was also reported for the UGTs UGT73C5 and UGT73C6, which glucosylate BRs, but in addition can also detoxify the *Fusarium* mycotoxin deoxynivalenol (34), an inhibitor of protein translation. In this context, it is interesting that according to the STRING v11 protein–protein interaction analysis tool (at *string-db.org*), *PMAT1* and *UGT73C6* are co-expressed (35). It is tempting to speculate that a co-regulation of *PMAT1* and *UGT73C5* and/or *UGT73C6* may contribute to an effective conversion of certain aglycon classes for rapid formation of malonyl-glucosides, which could be of relevance for BRs, but also for other acceptors including fungal toxins.

How such dual roles in phase II detoxification reactions and endogenous compound conversion are coordinated is a question that needs to be answered for various classes of phase II catabolic enzymes. One option may be an inducible expression by the acceptor or upstream precursors, and there is first evidence that *PMAT1* is BL-induced. Another mode is a cell-type specific expression or developmental regulation, and publicly available microarray data suggest that *PMAT1* transcript, while being present in all organs, is most abundant in seeds and flowers (Arabidopsis eFP browser at *bar.utoronto.ca*; (36)). Therefore, it is clear that further studies are needed to dissect the seemingly unrelated roles of PMAT1 in BR homeostasis and phase II detoxification processes and elucidate the physiological processes it specifically contributes to.

## Experimental procedures

### Plant material and growth conditions

*A. thaliana* (L.) Heynh. ecotype Columbia-0 (Col-0) was the WT background of all lines used. The T-DNA insertion lines *at5mat-2* (SM_3_35,619; NASC stock number N122330) and *pmat1-2* (SALK_007564; NASC stock number N507564) were obtained from NASC. The sites of insertion were mapped by PCR and sequencing (all primers used in this study are listed in Table S2). To generate the *pmat1 at5mat* double knock-out mutant, *at5mat-2* and *pmat1-2* single mutant plants were crossed, the F2 offspring genotyped with genotyping primers and homozygous F3 plants were selected. For generation of *At5MAT* and *PMAT1* overexpression lines, the ORFs of the two genes were cloned into the plant expression vector pGWR8 (37) as described in Table S3. Plants were transformed with the floral-dip method (38), and homozygous lines from independent transgenic individuals were selected. To generate double overexpressors, the lines *35S:PMAToe#8* or *35S:At5MAToe#10* were crossed with line *35S::UGT73C6:YFP-30* (6), and homozygous double overexpressors were selected by genotyping. Transgene abundance was quantified by qPCR.

In general, plants were cultivated in BrightBoy growth chambers (CLF Plant Climatics, Wertingen, Germany) in long days (16 h 80 μmol m^-2^ s^-1^ white light/8 h dark) and at a temperature of 21 °C ±2 °C. They were grown either in sterile conditions on half-strength Murashige and Skoog (½ MS) medium (39) (with 1% sucrose and 0.8% plant agar) or in soil (SP ED63P, Patzer GmbH, Sinntal-Altengronau). For hypocotyl elongation assays, seeds were plated on ½ MS plates containing 24-epiBL or solvent (dimethyl sulfoxide) in equal quantities as a control, stratified for 48 h at 4 °C, and then incubated vertically in the light or in the dark (with a prior 6 h light impulse). The length of the hypocotyls of plants germinated at the same time was then measured at different time points. For adult plant phenotyping, plants were pregrown on ½ MS medium and transferred to soil at 5 days post germination.

### Wheat germ extract–based *in vitro* translation and recombinant protein production

All cloning procedures are described in detail in Table S2. For *in vitro* protein translation, constructs that contain the coding sequences of the selected acyltransferases in the pUC SP6 vector were generated. The *in vitro* translation was performed according to the manufacturer instructions with the TNT SP6 High-Yield Wheat Germ Master Mix (Promega, Cat. L3261). The reactions were incubated at 25 °C for 2 h prior addition of 25 μl glycerol for storage at −20 °C. Translation products were analyzed by Western blot analysis.

For production of recombinant GST-fusion proteins, *PMAT1* and *At5MAT* were cloned in frame into pGEX vectors (GE Healthcare, cat. 28–9545–50) to obtain pGEX-4T-2-PMAT1 or pGEX-4T-2-At5MAT (for details see Table S2). The plasmids were then transformed into *E. coli* BL21, and protein expression was induced as described previously (40). Recombinant proteins were purified using Glutathione Sepharose 4B beads (GE Healthcare, cat. 17–0756–01) according to the recommendations of the manufacturer.

### *In vitro* malonylation assays

For *in vitro* malonylation assays, 5 μl of *in vitro* translated proteins were mixed with 10 μl assay buffer containing 0.5 μM epiBL-23-*O*-Glc, 2.5 mM malonyl-CoA, 100 mM diethanolamine/HCl pH 9.0, and 20 mM DTT, and water was added to a total reaction volume of 20 μl. The tubes were incubated at 30 °C overnight, and the reactions were stopped by adding 10 μl 10% trichloroacetic acid and analyzed by TLC.

For enzyme assays with recombinant protein, the reaction conditions were the same; however, either different amounts of purified GST-PMAT1 and GST-At5MAT (3, 1.5, 0.75, 0.375, 0.18, 0.09, 0.045, 0.0225, 0.01, 0.005 μg in 5 μl) or buffers with different pH values (50 mM diethanolamine set with HCl to pH 6–10.5) were used. All reaction products were analyzed by TLC.

For enzyme kinetic studies, reactions were performed in the same way except that 50 mM sodium phosphate buffer pH 8.0 was used, and the substrate was added to obtain the concentrations indicated in Figure 1*B*. In case of PAMT1, 5.2 ng of GST fusion protein was used, and the reactions were incubated at 30 °C for 20 min. For At5MAT, 46 ng of GST fusion protein was used, and the reactions were incubated at 30 °C for 240 min. Analysis by TLC and quantification was performed as described below.

### TLC

Reaction products were extracted twice with 100 μl ethyl acetate. The combined organic extracts were evaporated in the vacuum. The residue was dissolved in 10 μl ethyl acetate and analyzed by TLC as described previously (11) except that the plates were developed in chloroform/ethyl acetate/methanol/formic acid/water (= 10/10/5/2/1). Subsequently, the plates were sprayed with 1% sulfuric acid in methanol and heated to 110 °C for 10 min. The fluorescent spots were visualized by excitation with UV of 366 nm and quantified using ImageJ.

### Western blot analysis

Five microliter of protein preparations produced in the wheat germ expression system were mixed with 5 μl two x SDS buffer (100 mM TRIS/HCl pH 6.8, 200 mM DTT, 4% SDS, 20% glycerol and 0.025% bromophenol blue) and heated to 95 °C for 3 min. Samples were separated on a 10% SDS-PAGE gel and transferred onto a PVDF membrane (Merck Millipore). After blocking with TBS-T (150 mM NaCl, 10 mM TRIS/HCl pH 8.0, 0.1% Tween 20) containing 5% skim milk powder, the membrane was probed with anti-c-Myc-HRP antibody (cat. sc40 HRP, Santa Cruz Biotechnology) diluted 1:5000 in TBS-T containing 5% skim milk powder. The membrane was washed six times in TBS-T and one time in phosphate buffered saline prior signal detection using the ECL Select kit (cat. RPN2235, GE Healthcare).

To probe BES1 phosphorylation, opened flowers of adult plants were frozen and ground to a fine powder in liquid nitrogen. Proteins were extracted by addition of two volumes two x SDS buffer and heating to 95 °C for 5 min. Extracts (7.5 μl) were separated on 15% SDS-PAGE gels and blotted and blocked as described above. The membrane was probed with a BES1 antibody (21) 1:2000 in TBS-T-containing 5% skim milk powder at 4 °C overnight. After washing six times with TBS-T containing 5% skim milk powder, the membrane was probed with HRP-conjugated anti-mouse antibody (cat. 61–6020, Invitrogen) diluted 1:10,000 in TBS-T containing 5% skim milk powder at 4 °C overnight. Final washing and signal detection was performed as stated above.

### qPCRs

RNA isolation, cDNA synthesis, and qPCR was performed as described previously (11). The amplification curves were linear (r^2^ > 0.99), and the primer pairs showed high efficiency (95–105%). Melting curves confirmed absence of unspecific by-products. Expression levels were normalized to the internal standard *GAPC2* and measured in three to four technical replicates. Primers used for qPCR are listed in Table S1.

## Data availability

All data are contained in the manuscript and the supporting information. Material that was generated is available upon request from the corresponding author Brigitte Poppenberger: brigitte.poppenberger@wzw.tum.de

## Supporting information

This article contains supporting information (1, 4, 5, 6).

## Conflict of interests

The authors declare that there is no conflict of interests.
