# Supplementary material for: The acyltransferase PMAT1 malonylates brassinolide glucoside
Source: J Biol Chem. 2021 Feb 16;296:100424. doi: 10.1016/j.jbc.2021.100424 (PMC8010461; doi:10.1016/j.jbc.2021.100424)
Supplement: Figures and Tables [file mmc1.pdf]

Supporting Information for

The acyltransferase PMAT1 malonylates brassinolide glucoside

Sufu Gan[a,1], Wilfried Rozhon[a,1], Elisabeth Varga[b,2], Jyotirmoy Halder[a,3], Franz Berthiller[b] and Brigitte Poppenberger[a,]*

This file contains:

SI Experimental procedures

Tables S1-S3

SI References

Figures S1-S11

SI Experimental procedures

Synthesis of 24-epiBL- (epiBL) and BL-glucoside. Recombinant GST-UGT73C5 protein was expressed in *E. coli* BL21 cells containing pGEX-4T-3-UGT73C5 (1) and purified in the same way as GST-PMAT1 and GST-At5MAT. For *in vitro* glucosylation reactions, 1 mg GST-UGT73C5 was added to 4 mL of a solution containing 0.5 mM epiBL, 10 mM UDP-glucose, 0.5 mM ATP, 50 mM TRIS/HCl pH 8.0, 50 mM $MgCl_2$ and 10 mM DTT and incubated at 30 °C overnight. The reaction products were purified by preparative HPLC using a Shimadzu LC-10Avp system equipped with a SPD-10Avp UV detector, a FRC-10A fraction collector and a Luna 5 µm 100 Å C18(2) 100 x 21 mm column (Phenomenex, Aschaffenburg, Germany). Elution was carried out using mobile phases A (10% acetonitrile in water) and B (100% acetonitrile). The gradient started with 0% B, which was linearly increased to 80% within 120 min at a constant flow rate of 2 mL/min. Chromatograms were recorded with UV detection at 200 nm. Fractions (4 mL) were collected and aliquots were analyzed by TLC. Fractions containing epiBL-23-*O*-Glc (retention time 82 min to 86 min) were pooled and evaporated to dryness using a vacuum concentrator. The obtained product was dissolved in DMSO and stored at -20°C. BL-23-*O*-Glc was synthesized and purified in the same way except that BL (instead of epiBL) was used as a substrate and the product was dissolved in ethyl acetate. The yields of epiBL-Glc and BL-Glc were estimated by TLC (see below), which revealed amounts of approximately 0.38 mg and 0.25 mg, respectively.

To verify the structure of the obtained products, samples were analyzed by HPLC-QTOF (see below) alongside with authentic BL-2-*O*-Glc, BL-3-*O*-Glc, BL-22-*O*-Glc and BL-23-*O*-Glc (2), which showed retention times of 8.54, 10.13, 10.02 and 8.68 min, respectively. The prepared BL-Glc had a retention time of 8.66 min, confirming that it is the 23-*O*-glucoside. The retention time of the prepared epiBL-Glc was 8.32 min (no authentic reference standard available for comparison). The MS spectra of all compounds and the synthesized products were virtually identical (see Figure S4 and S5 for the MS and MS/MS spectra of the synthesized compounds). As it is expected that UGT73C5 utilizes the same hydroxy groups in BL and epiBL the obtained epiBL reaction product was tentatively identified as epiBL-23-*O*-Glc.

Synthesis of epiBL- and BL-malonylglucosides. Recombinant GST-PMAT1 protein (10 µg) was added to 1.2 mL of a mixture containing 50 mM diethanolamine/HCl pH 7.5, 1.25 mM malonyl-CoA, 10 mM DTT and approximately 0.15 mg BL-23-*O*-Glc. The reactions were incubated at 30 °C overnight. The reaction products were separated by preparative HPLC using the system described above. Elution was carried out using mobile phases A (10 mM formic acid in 10% acetonitrile) and B (100% acetonitrile). The gradient started with 0% B, which was linearly increased to 50% B within 120 min at a constant flow rate of 2 mL/min. Fractions (4 mL) were collected and aliquots were analyzed by TLC (see below). Fractions containing BL-23-*O*-MalGlc (retention time 52 min to 54 min) were pooled and evaporated to dryness using a vacuum concentrator. The obtained product was dissolved in ethyl acetate and stored at -20 °C. The yield was estimated by TLC (see below), which indicated an amount of BL-23-*O*-MalGlc of approximately 0.086 mg. The procedure was repeated with epiBL-23-*O*-Glc to obtain epiBL-23-*O*-MalGlc.

Analysis by HPLC-QTOF revealed the expected mass for BL-23-*O*-MalGlc. In addition, the loss of the malonyl glucose residue as a whole (fragment 231.0 corresponds to $[MalGlc\text{-}2H_2O+H]^+$) indicates that the malonyl residue is attached to the glucose residue rather than the aglycon (Figure S6). The MS and MS/MS spectra of the proposed epiBL-23-*O*-MalGlc looks extremely similar (Figure S7), but the compounds can be differentiated according to their retention times.

Estimation of BL-23-*O*-Glc and BL-23-*O*-MalGlc concentrations. Since authentic standards with known concentrations are not available for epiBL-Glc, BL-23-*O*-Glc and particularly epiBL-23-*O*-MalGlc, we used BL standards for calibration assuming that treatment with sulfuric acid yields products with the same fluorescent spectral properties. In brief, samples were loaded alongside with BL standards (200, 400, 800, 1200, 2400, 3600 ng BL) on a TLC plate, which was developed and evaluated as described in the materials and methods section. Spot volumes were quantified using the Image J software. The obtained linear fit (R^2

= 0.98) allowed the estimation of the concentrations of BL-23-*O*-Glc and BL-23-*O*-MalGlc solutions, which were found to be 12.5 ng/µL and 14.3 ng/µL, respectively. These solutions were used as calibrants for HPLC-QTOF analysis.

BL Feeding Assays. For quantification of BL-23-*O*-MalGlc in seedlings 30-35 eleven-day-old seedlings of wild-type, *at5mat-2*, *pmat1-2*, *at5mat pmat1*, *35S:At5MAToe*#1, #5 and *35S:PMAT1oe*#3, #6, grown on ½ MS plates, were transferred to sterile flasks containing 30 mL liquid ½ MS medium. BL was added to a final concentration of 1 µg/mL and the flasks were incubated with gentle shaking (60 rpm) under continuous light (80 $\mu mol \cdot m^{-2} \cdot s^{-1}$) at 21°C for 48 h. The plant material was harvested, ground in liquid nitrogen and stored at -80 °C. Plant material (10-20 mg) was extracted twice with 100 µL of methanol/water = 1/1. For analysis of BL modification in flowers, opened flower buds from wild type, *35S:PMAT1oe*#8, *UGT73C6:YFP#30* and *PMAT1oe x UGT73C6oe* were transferred to tubes containing liquid ½ MS medium. BL was added to a final concentration of 1 µg/mL. Samples were incubated and extracted in the same way as described above.

High performance liquid chromatography – quadrupole time-of-flight mass spectrometry (HPLC-QTOF) analyses. Liquid chromatography high resolution mass spectrometry (LC-HR-MS) was used to quantify the brassinosteroid metabolites. Furthermore, LC-HR-MS/MS analyses were performed for structural elucidation. An Agilent 1290 UHPLC system was coupled with an Agilent 6550 iFunnel QTOF LC-MS instrument (Agilent, Santa Clara, CA, USA) and operated at the same conditions as shown recently (3). Briefly, analytes were separated on an Agilent Zorbax SB-C18 (150 x 2.1 mm, 1.8 µm particle size) column using gradient elution with water and methanol (both solvents containing 0.1% formic acid and 1 mM ammonium formate). Electrospray ionization in positive ion mode was performed with the following conditions: capillary voltage +4000 V, drying gas temperature 130 °C and drying gas flow rate 14 L/min, sheath gas temperature 300°C and sheath gas flow rate 10 L/min and nebulizer 30 psig. The QTOF was operated in the Extended Dynamic Range (2 GHz) mode obtaining three spectra. High mass accuracy was ensured by constantly infusing two reference compounds (*m/z* 121.0508 and 922.0098) via a second nebulizer. Data was acquired using MassHunter Workstation LC/MS Data Acquisition version B.08.00 and evaluated using MassHunter Qualitative Analysis versions B.07.00 and B.10.0.

SI Tables

Table S1. Kinetics of recombinant PMAT1 and At5MAT.

Protein	Tag	Substrate	K_m	k_{cat}	k_{cat}/K_m	Reference
PMAT1	GST	epiBL-Glc	235 μmol×L^{-1}	242 s^{-1}	1.0×10^{6} L×mol^{-1}×s^{-1}	This study
PMAT1	His$_6$	Kaempferol-7-O-glucoside	31 μmol×L^{-1}	33 s^{-1}	1.1×10^{6} L×mol^{-1}×s^{-1}	(4)
PMAT1	His$_6$	1-Naphthol glucoside (1-NAG)	160 μmol×L^{-1}	46 s^{-1}	2.9×10^{5} L×mol^{-1}×s^{-1}	(4)
PMAT1	His$_6$	2-Naphthol glucoside (2-NAG)	85 μmol×L^{-1}	40 s^{-1}	4.7×10^{5} L×mol^{-1}×s^{-1}	(4)
PMAT1	His$_6$	4-Nitrophenylglucoside	1600 μmol×L^{-1}	120 s^{-1}	7.5×10^{4} L×mol^{-1}×s^{-1}	(4)
PMAT1	His$_6$	4-Methylumbeliferylglucoside	550 μmol×L^{-1}	19 s^{-1}	3.5×10^{4} L×mol^{-1}×s^{-1}	(4)
At5MAT	GST	epiBL-Glc	71 μmol×L^{-1}	0.69 s^{-1}	9.7×10^{3} L×mol^{-1}×s^{-1}	This study
At5MAT	S-Tag	Cyanidin-3,5-diglucoside	6.6 μmol×L^{-1}	6.4 s^{-1}	9.7×10^{5} L×mol^{-1}×s^{-1}	(5)
At5MAT	S-Tag	Pelargonidin-3,5-diglucoside	4.5 μmol×L^{-1}	5.9 s^{-1}	1.3×10^{6} L×mol^{-1}×s^{-1}	(5)
At5MAT	S-Tag	Peonidin-3,5-diglucoside	6.9 μmol×L^{-1}	6.6 s^{-1}	9.6×10^{5} L×mol^{-1}×s^{-1}	(5)

Table S2. Oligonucleotides used in this study.

Primer name	Sequence (restriction sites used for cloning are underlined)	Purpose
PMAT1 fwd	CACACCATGGTGAACGAAGAAATGGAG	Cloning to pGEX-4T-2
PMAT1 rev	AAAAGCGGCCGCTATTTTCTAGTCCCTTGTGAAG	Cloning to pGEX-4T-2
At5MAT fwd	TCAGCCATGGTGAATTTCAACTCAGCCG	Cloning to pGEX-4T-2
At5MAT rev	GTTCGCGGCCGCTTTCCAACCCGATGGAGAATA	Cloning to pGEX-4T-2
At3g29680 fwd	TCAACCATGGCCTTAAACGTGATCAAGATC	Cloning to pGEX-4T-2
At3g29680 rev	ATAGGCGGCCGCTTTTGTAGATATCGAAATCACTGG	Cloning to pGEX-4T-2
At5g39080 fwd	GGAGACATGTTTTCATCACTTAACATCATC	Cloning to pGEM-T Easy
At5g39080 rev	AAAAGCGGCCGCTAATAGTTAGTTCACAATGAAGCA	Cloning to pGEM-T Easy
At3g29635 fwd	CATACATGTCGCTAAAGGTAACCAAGATCTC	Cloning to pGEM-T Easy
At3g29635 rev	ATCAGCGGCCGCTAAGAACCATTTCTAAACCATTT	Cloning to pGEM-T Easy
At5g61160 fwd	TGAGACATGTCGTTAAAGGTGATCAAGAT	Cloning to pGEM-T Easy
At5g61160 rev	CCGTGCGGCCGCTGTTTCCAATCCATTTGTAAAAT	Cloning to pGEM-T Easy
PMAT2 fwd	TCACTCATGACGCTACACGTCATTGAGAC	Cloning to pGEM-T Easy
PMAT2 rev	AAACGCGGCCGCTATGTAAACCATTGTTGAAAAA	Cloning to pGEM-T Easy
At5g39090 fwd	AAAATCATGAATCCATCACTAAACTTCATC	Cloning to pGEM-T Easy
At5g39090 rev	TGTTGCGGCCGCTTTTTATCCCCTTGTGTAGCAAA	Cloning to pGEM-T Easy
PMAT1 ko fwd	TCTCATAACACATCATGGTGAACGA	Genotyping/ RT-PCR
PMAT1 ko rev	TCCGCAAAAGAAATCGCTTCA	Genotyping/ RT-PCR
SalkLBb1	GCGTGGACCGCTTGCTGCAACT	Genotyping
At5MAT ko fwd	TTAAACCCTCCGTTACCGCCGAC	Genotyping/ RT-PCR
At5MAT ko rev	GGTCAAAGTCTCCACCGCCG	Genotyping/ RT-PCR
dSpm LB	TACGAATAAGAGCGTCCATTTTAGAGT	Genotyping
At5g39090 fwd	CATGCCATGGATCCATCACTAAACTTCATC	Transfer of At5g39090
At5g39090 rev	ATAAGAATGCGGCCGCTTTTTATCCCCTTGTGTAGCAAA	Transfer of At5g39090
PMAT1 fwd	TTTACGGGTTGGATTTTGGGTG	qPCR
PMAT1 rev	TCCGCAAAAGAAATCGCTTCA	qPCR
At5MAT fwd	TAGGGATTTACGGGTCTGATTT	qPCR
At5MAT rev	GAAACCGAAGCATCCTTATCAA	qPCR
GAPC2 fwd	TTGGTGACAACAGGTCAAGCA	qPCR/ RT-PCR
GAPC2 rev	AAACTTGTCGCTCAATGCAATC	qPCR/ RT-PCR
CPD-1	CTTGCTCAACTCAAGGAAGAG	qPCR
CPD-2	CTCGTAGCGTCTCATTAACCAC	qPCR
BR6ox2-1	AGCTTGTTGTGGGAACTCTATCGG	qPCR
BR6ox2-2	CGATGTTGTTTCTTGCTTGGACTC	qPCR
ROT3-3	CTTGTAACCCGGTACAGTTGC	qPCR
ROT3-4	TCCGCTTCATCTTCACAGTC	qPCR
UGT73C6-fwd	GTAAGTGCCGAGGTTAAAGAGG	qPCR
UGT73C6q-rev	TCTCCAAGCTCTTTGGCTCT	qPCR

Table S3. Vectors and constructs used in this study.

Plasmids	Description/cloning	Source or reference
pGEX-4T-2	Expression vector for *E. coli*	GE Healthcare, cat. 28-9545-50
pGEM-T Easy	Cloning vector	Promega, cat. A1380
pGWR8	pGreen-based plant expression vector	(6)
pGEX-4T-3-UGT73C5	Construct for expression of UGT73C5 (DOGT1) in *E. coli*. The construct was originally named pGEX-4T-3-DOGT1.	(1)
pGEX-4T-2-PMAT1	The CDS was amplified from wild type gDNA using the primer pair PMAT1 fwd/PMAT1 rev. The obtained PCR product was digested with NcoI/NotI and cloned into pGEX-4T-2 linearized with StyI/NotI.	This study
pGEX-4T-2-At5MAT	The CDS was amplified from wild type gDNA using the primer pair At5MAT fwd/At5MAT rev. The obtained PCR product was digested with NcoI/NotI and cloned into pGEX-4T-2 linearized with StyI/NotI.	This study
pGEX-4T-2-At3g29680	The CDS was amplified from wild type gDNA using the primer pair At3g29680 fwd/At3g29680 rev. The obtained PCR product was digested with NcoI/NotI and cloned into StyI/NotI-linearized pGEX-4T-2.	This study
pGEM-T Easy-At5g39080	The CDS was amplified from wild type gDNA using the primer pair At5g39080 fwd/At5g39080 rev. The obtained amplicon was TA-cloned into pGEM-T Easy.	This study
pGEM-T Easy -At3g29635	The CDS was amplified from wild type gDNA using the primer pair At3g29635 fwd/At3g29635 rev. The obtained amplicon was TA-cloned into pGEM-T Easy.	This study
pGEM-T Easy -At5g61160	The CDS was amplified from wild type gDNA using the primer pair At3g29680 fwd/At3g29680 rev. The obtained amplicon was TA-cloned into pGEM-T Easy.	This study
pGEM-T Easy -PMAT2	The CDS was amplified from wild type gDNA using the primer pair PMAT2 fwd/PMAT2 rev. The obtained amplicon was TA-cloned into pGEM-T Easy.	This study
pGEM-T Easy -At5g39090	The CDS was amplified from wild type gDNA using the primer pair At5g39090 fwd/At5g39090 rev. The obtained amplicon was TA-cloned into pGEM-T Easy.	This study
pUC SP6	A synthetic DNA fragment (see Figure S11) was digested with EcoRI/HindIII and cloned in pUC18 digested with the same enzymes. The construct contains the elements required for expression of proteins using the wheat germ extract translation system. The obtained proteins contain a C-terminal Myc-tag.	This study
pUC SP6-PMAT1	The NcoI/NotI restriction fragment of pGEX-4T-2-PMAT1 was cloned into pUC SP6 linearized with NcoI/NotI.	This study
pUC SP6-At5MAT	The NcoI/NotI restriction fragment of pGEX-4T-2-At5MAT was cloned into pUC SP6 linearized with NcoI/NotI.	This study
pUC SP6-At3g29680	The NcoI/NotI restriction fragment of pGEX-4T-2- At3g29680 was cloned into pUC SP6 linearized with NcoI/NotI.	This study
pUC SP6-At5g39080	The PciI/NotI restriction fragment of pGEM-T Easy-At5g39080 was cloned into pUC SP6 linearized with NcoI/NotI.	This study
pUC SP6-At3g29635	The PciI/NotI restriction fragment of pGEM-T Easy - At3g29635 was cloned into pUC SP6 linearized with NcoI/NotI	This study
pUC SP6-At5g61160	The PciI/NotI restriction fragment of pGEM-T Easy - At5g61160 was cloned into pUC SP6 linearized with NcoI/NotI.	This study
pUC SP6-PMAT2	The BspHI/NotI restriction fragment of pGEM-T Easy - PMAT2 was cloned into pUC SP6 linearized with NcoI/NotI.	This study
pUC SP6-At5g39090	The amplicon obtained with primer pair At5g39050 fwd and At5g39090 rev and pGEM t easy-At5g39090 as template was restriction with BspHI /NotI and cloned into pUC SP6 linearized with NcoI/NotI.	This study
pGWR8-35S:PMAT1	The NcoI/NotI restriction fragment of pGEX-4T-2-PMAT1 was cloned into pGWR8 linearized with NcoI/NotI.	This study
pGWR8-35S:At5MAT	The NcoI/NotI restriction fragment of pGEX-4T-2-At5MAT was cloned into pGWR8 linearized with NcoI/NotI.	This study

SI References

1. Poppenberger, B., Berthiller, F., Lucyshyn, D., Sieberer, T., Schuhmacher, R., Krska, R. Kuchler, K., Glössl, J., Luschnig, C., and Adam, G. (2003) Detoxification of the Fusarium mycotoxin deoxynivalenol by a UDP glucosyltransferase from *Arabidopsis thaliana*. *J. Biol. Chem*. **278**, 47905-47914
2. Poppenberger, B., Fujioka, S., Soeno, K., George, G. L., Vaistij, F. E., Hiranuma, S., Seto, H., Takatsuto, S., Adam, G., Yoshida, S., and Bowles, D. (2006) The UGT73C5 of *Arabidopsis thaliana* glucosylates brassinosteroids. *Proceedings of the National Academy of Sciences*, **102**, 15253-15258
3. Gan, S., Rozhon, W., Varga, E., Unterholzner, S. J., Berthiller, F., and Poppenberger, B. (2020) The BAHD acyltransferase BIA1 uses acetyl-CoA for catabolic inactivation of brassinosteroids. *Plant Physiol*. **184**, 23-26
4. Taguchi, G., Ubukata, T., Nozue, H., Kobayashi, Y., Takahi, M., Yamamoto, H., and Hayashida, N. (2010) Malonylation is a key reaction in the metabolism of xenobiotic phenolic glucosides in Arabidopsis and tobacco. *Plant J*. **62**, 215-223
5. Luo, J., Nishiyama, Y., Fuell, C., Taguchi, G., Elliott, K., Hill, L., Tanaka, Y., Kitayama, M., Yamazaki, M., Bailey, P., Barr, A., Michael, A. J., Saito, K., and Martin, C. (2007) Convergent evolution in the BAHD family of acyl transferases: identification and characterization of anthocyanin acyl transferases from *Arabidopsis thaliana*. *Plant J*. **50**, 678-695
6. Rozhon, W., Mayerhofer, J., Petutschnig, E., Fujioka, S., and Jonak, C. (2010) ASKθ, a group-III Arabidopsis GSK3, functions in the brassinosteroid signalling pathway. *Plant J*. **62**, 215-223

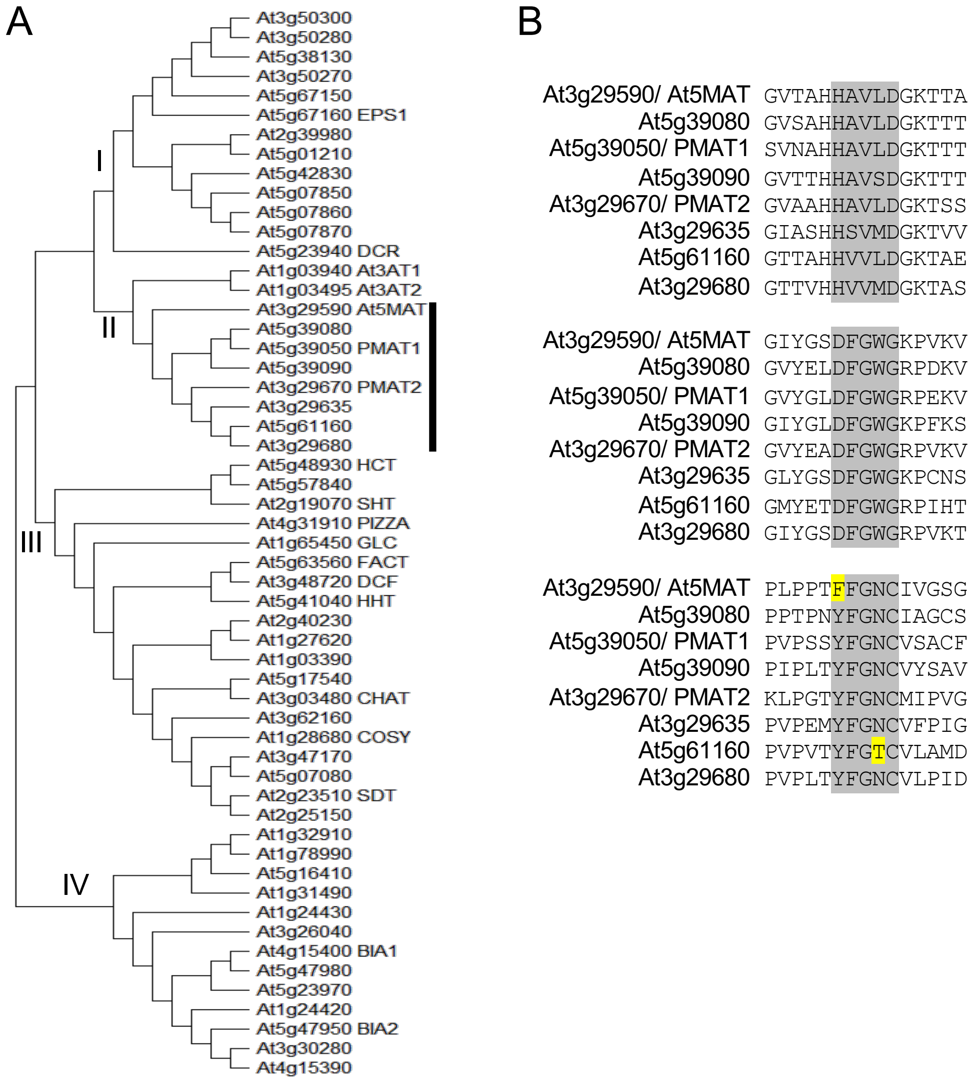

Figure S1. Identification of putative malonyltransferases in the BAHD acyltransferase family of *A. thaliana*.

(*A*) Phylogenetic tree of 55 BAHD acyltransferases of *A. thaliana* that were identified with BLAST searches using the amino acid sequence of PMAT1 as a bait. The MEGA (version 10.0.5) software was utilized for tree assembly by neighbor joining with default parameter settings. Locus identifiers, and, if known, gene names are given. The candidates chosen for an *in vitro* characterization are marked with a black line. (*B*) Alignment of BAHD signature domain-containing areas of the clade II acyltransferases selected for characterization.

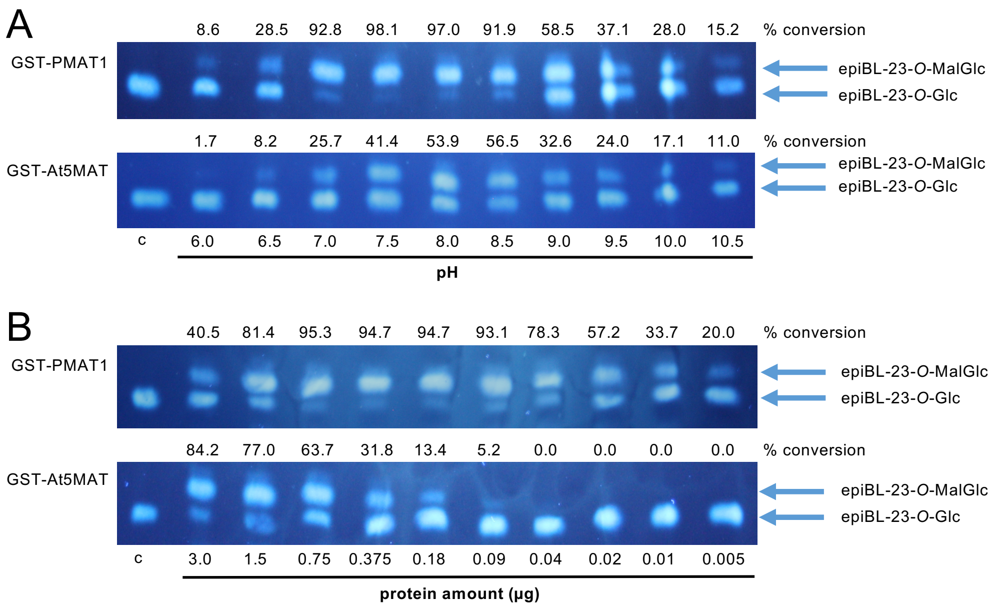

Figure S2. Characterization of pH and input protein optima for *in vitro* activity of PMAT1 and At5MAT.

In vitro malonylation reactions were carried out with recombinant, GST-tagged PMAT1 or At5MAT, epiBL-23-*O*-Glc as an acceptor and malonyl-CoA as a donor substrate. The reaction products were separated by TLC and visualized with UV light (366 nm). Spot intensities were quantified with ImageJ and the conversion rate, defined as the spot intensity of the product divided by the sum of the spot intensities of the precursor and the product, are shown in % above the graphs. (A) Reaction buffers with different pH values and 180 ng of GST-PMAT1 or 750 ng of GST-At5MAT protein were used. (B) Different input protein amounts, in a reaction buffer with pH 8.0 were tested.

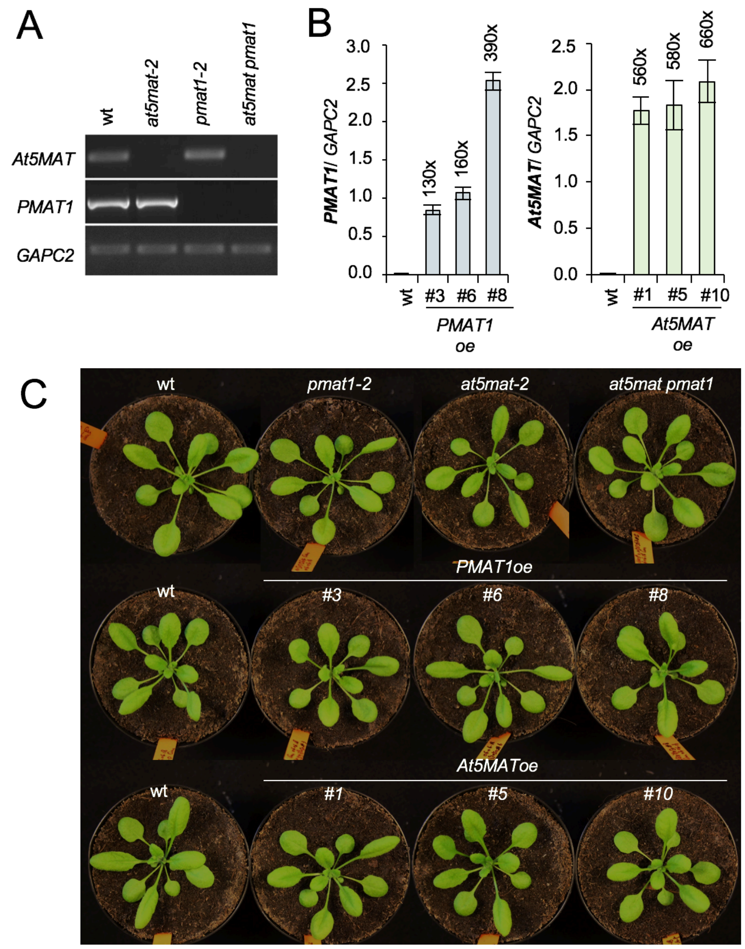

Figure S3. Generation of a double *at5mat pmat1* knock-out and *PMAT1* and *At5MAT* over-expressing plants.

(*A*) Semi-quantitative PCRs with gene specific primers, to determine transcript abundance in the knock-outs. *GAPC2* was amplified as an internal control. (*B*) Expression levels of *PMAT1* or *At5MAT* in over-expression lines relative to wild-type. qPCRs were performed from eleven-day-old plants grown on ½ MS medium, using gene specific primers and *GAPC2* for normalization. The average and SD of three biological replicates measured in four technical repeats is shown. (*C*) Photos of representative plants of each line used, grown for 3 weeks in LDs at 80 µmol·m^{-2}·s^{-1} of white light and 21°C. Multiple images were made into a digital composite for comparison.

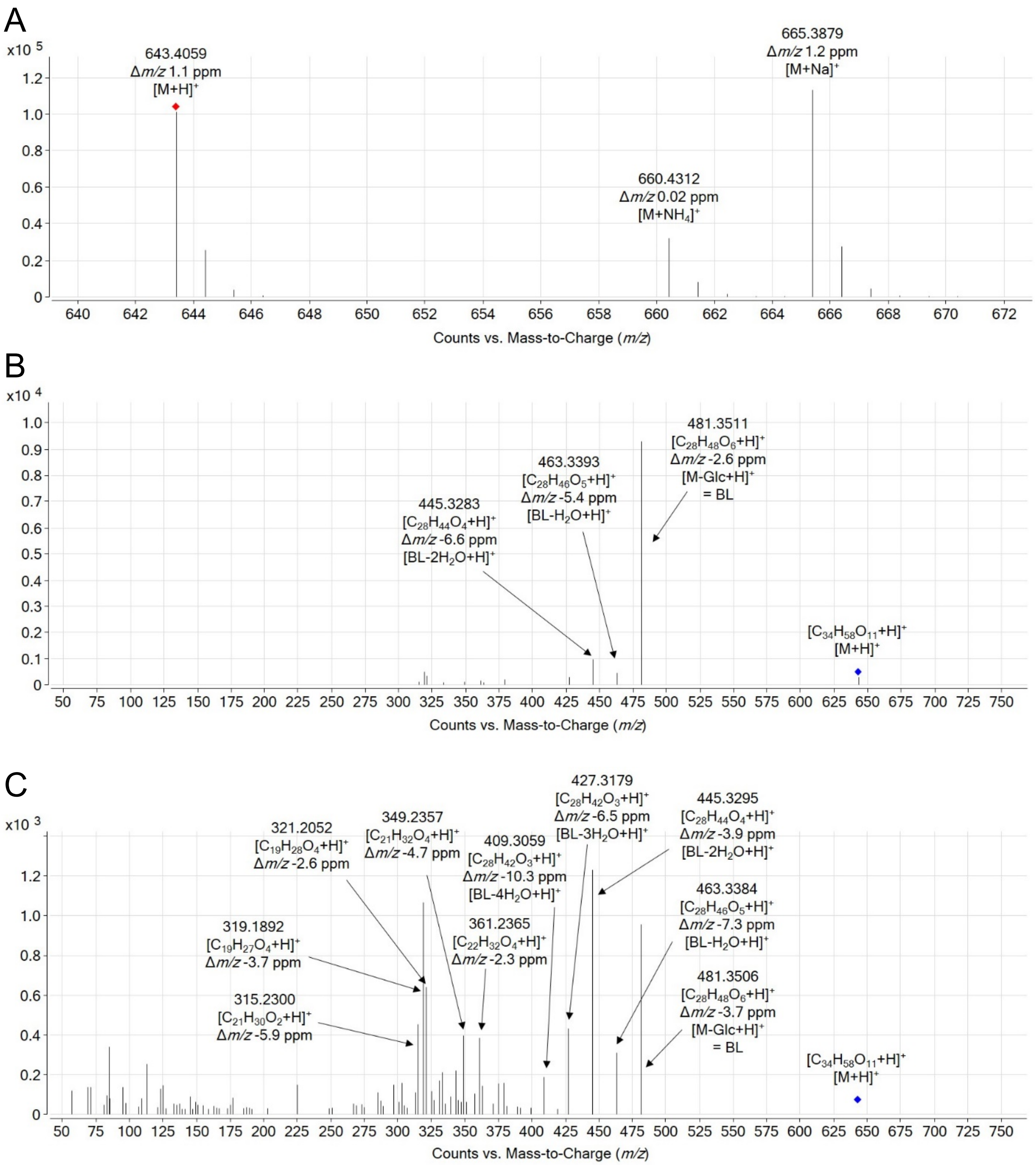

Figure S4. LC-HR-MS and LC-HR-MS/MS spectra of the protonated ion species of BL-23-*O*-glucoside at a retention time of 8.66 min.

(*A*) LC-HR-MS spectrum. (*B*) LC-HR-MS/MS spectrum at a collision energy of 10 eV. (*C*) LC-HR-MS/MS spectrum at a collision energy of 20 eV.

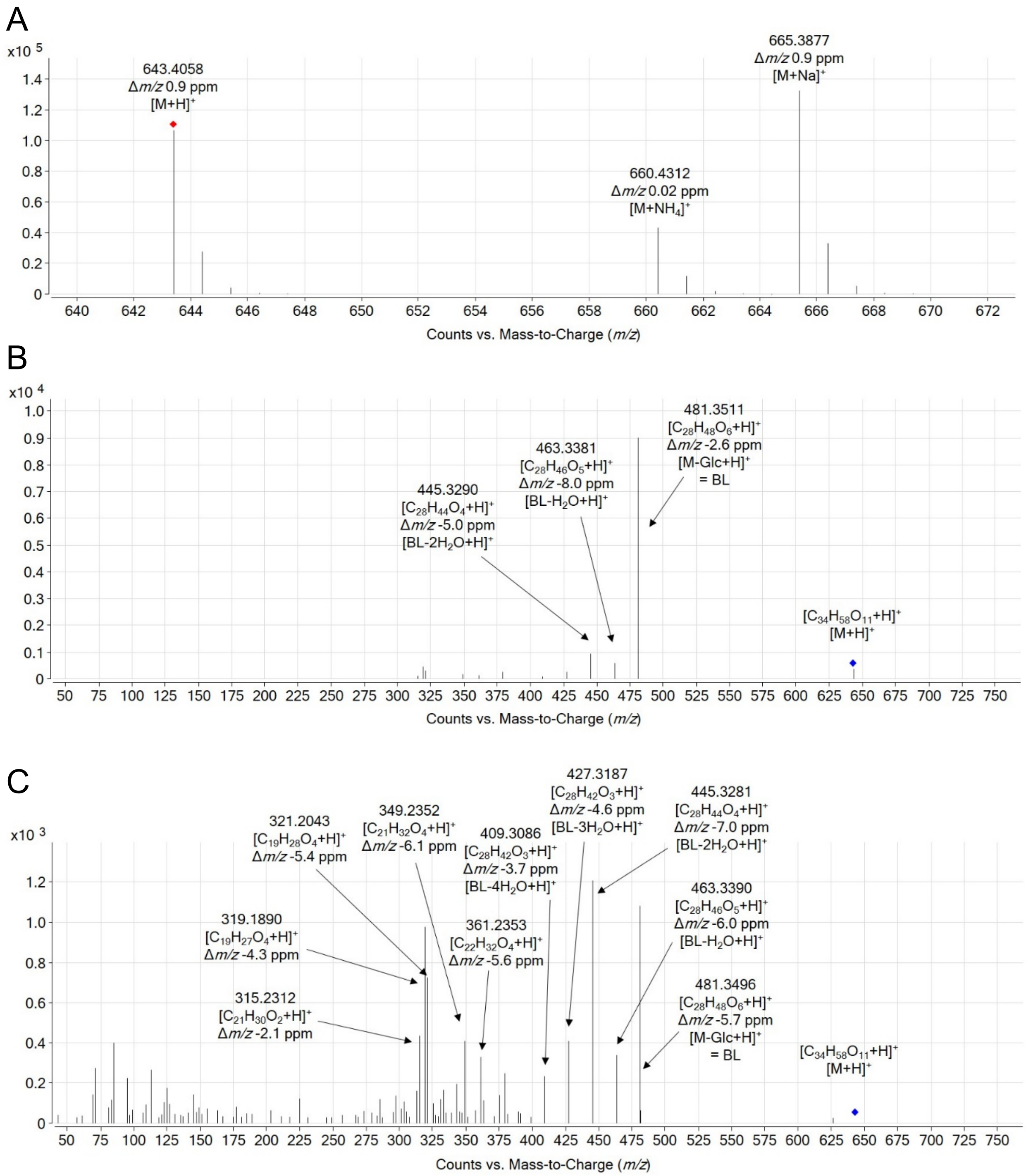

Figure S5. LC-HR-MS and LC-HR-MS/MS spectra of the protonated ion species of epiBL-23-*O*-glucoside at a retention time of 8.32 min.

(*A*) LC-HR-MS spectrum. (*B*) LC-HR-MS/MS spectrum at a collision energy of 10 eV. (*C*) LC-HR-MS/MS spectrum at a collision energy of 20 eV.

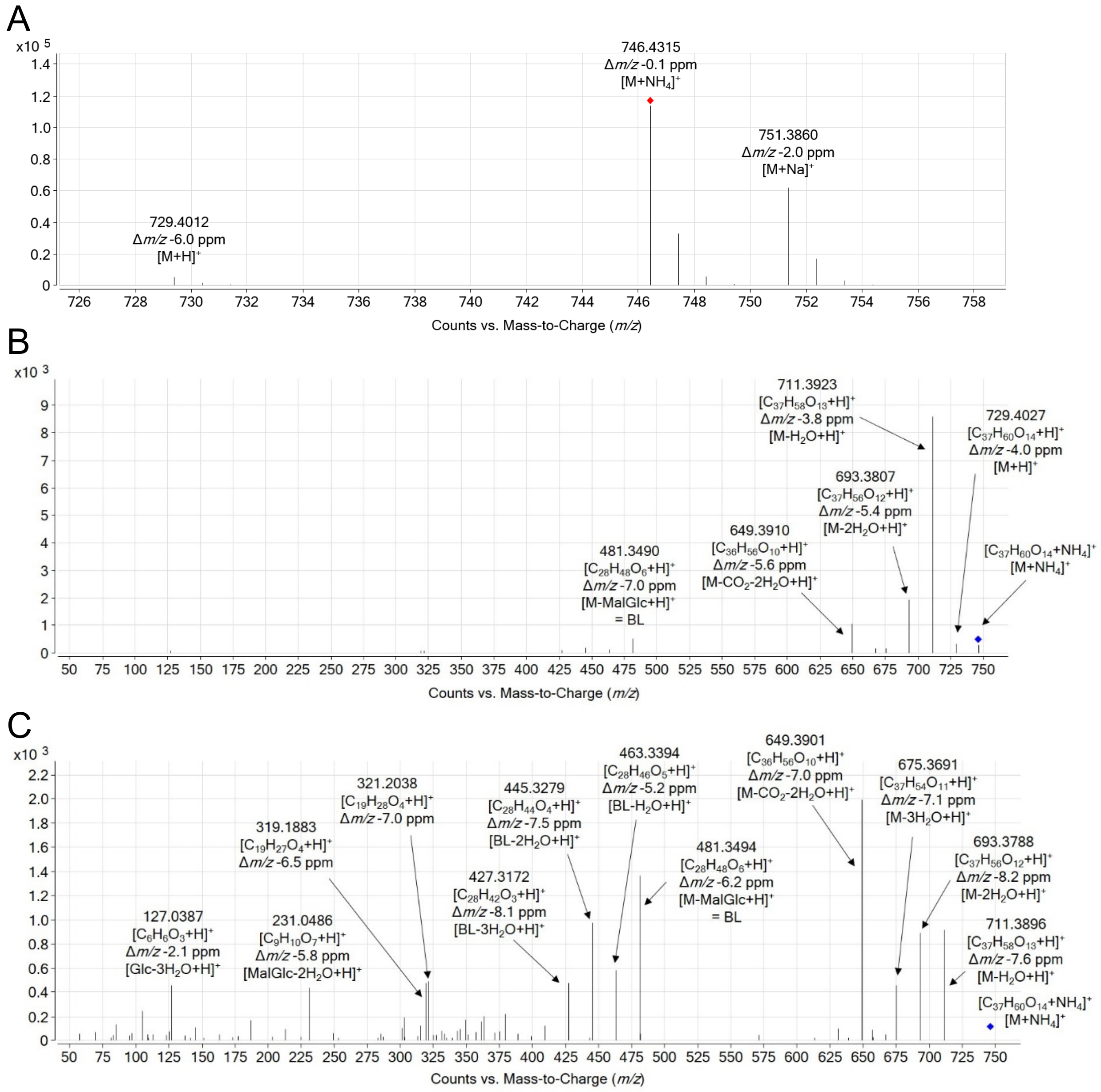

Figure S6. LC-HR-MS and LC-HR-MS/MS spectra of the ammonium adduct of BL-23-*O*-malonyl-glucoside at a retention time of 8.78 min.

(*A*) LC-HR-MS spectrum. (*B*) LC-HR-MS/MS spectrum at a collision energy of 10 eV. (*C*) LC-HR-MS/MS spectrum at a collision energy of 20 eV.

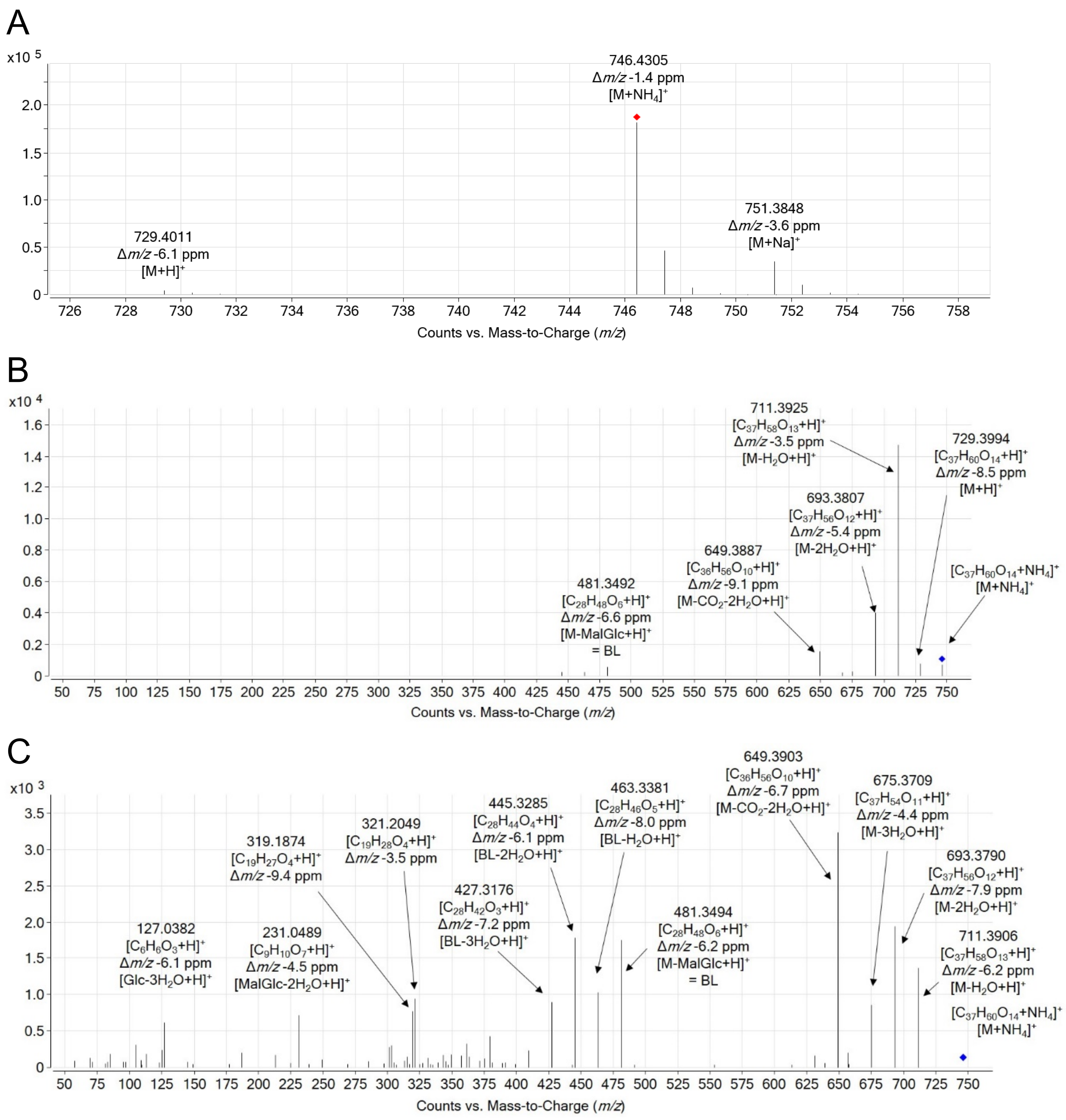

Figure S7. LC-HR-MS and LC-HR-MS/MS spectra of the ammonium adduct of epiBL-23-*O*-malonyl-glucoside at a retention time of 8.38 min.

(*A*) LC-HR-MS spectrum. (*B*) LC-HR-MS/MS spectrum at a collision energy of 10 eV. (*C*) LC-HR-MS/MS spectrum at a collision energy of 20 eV.

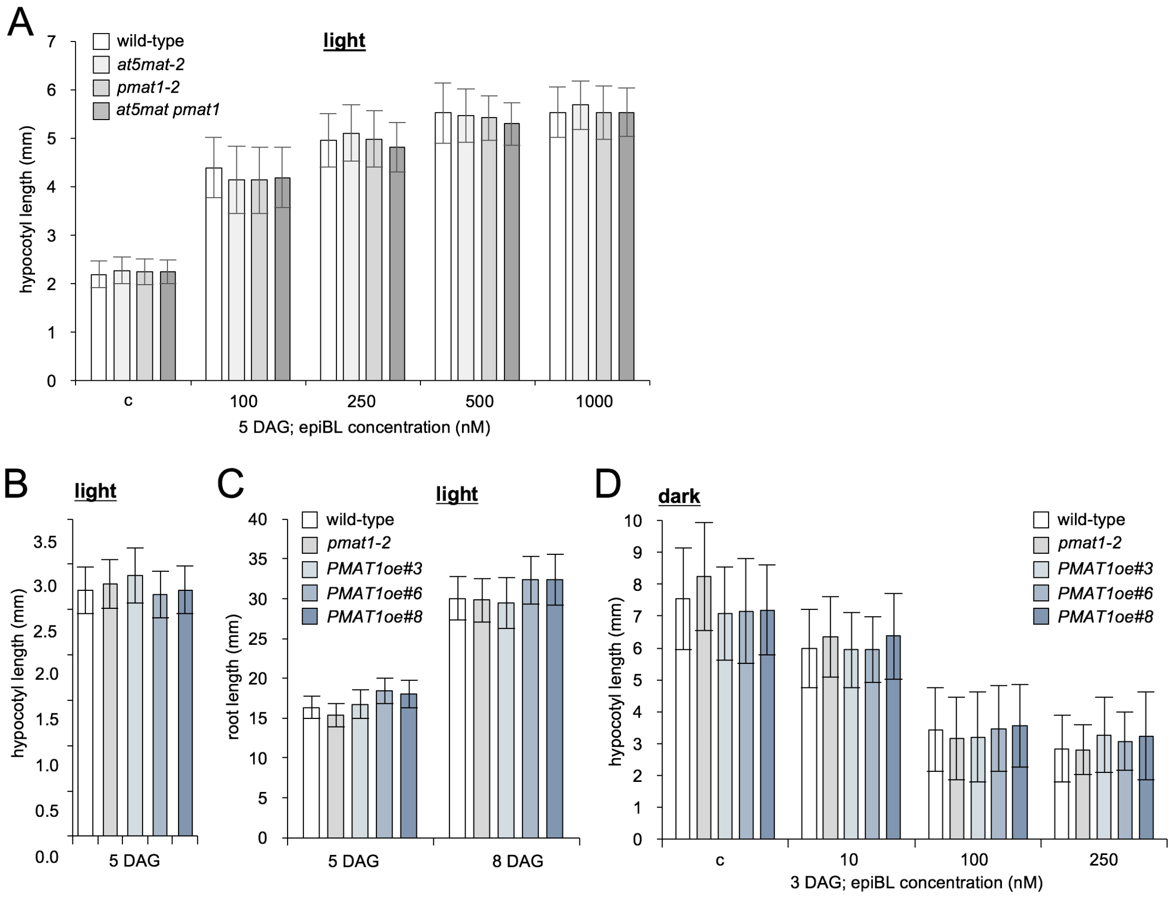

Figure S8. Altering *PMAT1* expression does not impact the development or BR-responsive growth of seedlings.

(*A*) epiBL response of light-grown seedlings of *at5mat-2* and *pmat1-2* single and double knock-out mutants. Seeds were germinated on ½ MS medium supplemented with 100, 250, 500, 1000 nM epiBL and grown for 5 days in LDs under low light (16 h white light, 20 μmol·m^{-2}·s^{-1}/8 h dark). Plates supplemented with DMSO served as control (c). The columns and bars show the average and SD of at least 65 measured hypocotyls. (*B*) Hypocotyl length and (*C*) primary root length of light-grown seedlings of the lines shown, grown for either 5 and/or 8 days in the same conditions as in A. (*D*) Hypocotyl length of dark-grown seedlings at 3 DAG. Seedlings were grown on ½ MS medium supplemented with 10, 100, 250 nM epiBL in the dark for 3 days. Plates supplemented with DMSO served as control (c). The columns and bars represent the average and SD of at least 25 individuals.

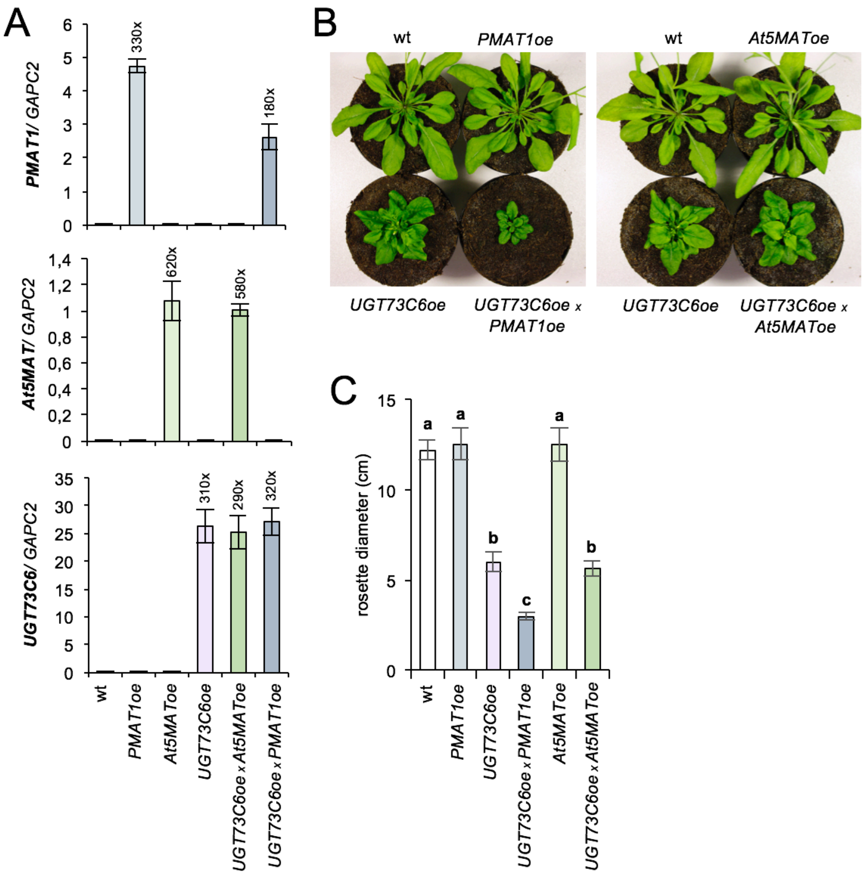

Figure S9. Characterization of *UGT73C6oexPMAT1oe* and *UGT73C6oexAt5MAToe* double over-expressors.

(*A*) Expression of *PMAT1*, *At5MAT* and *UGT73C6* in seedlings of the over-expression lines relative to wild-type (wt). qPCRs were performed from eleven-day-old plants grown on ½ MS medium, using gene specific primers and *GAPC2* for normalization. The average and SD of 3-4 biological replicates measured in 4 technical repeats is shown. (*B*) Phenotypic evaluation of adult *UGT73C6oexPMAT1oe* and *UGT73C6oexAt5MAToe* plants. Photos of 5-week-old, representative plants grown in LDs at 80 µmol·m^{-2}·s^{-1} of white light and 21°C. (*C*) Rosette diameter of the plants shown in B. Values are the means and SD of at least 12 plants.

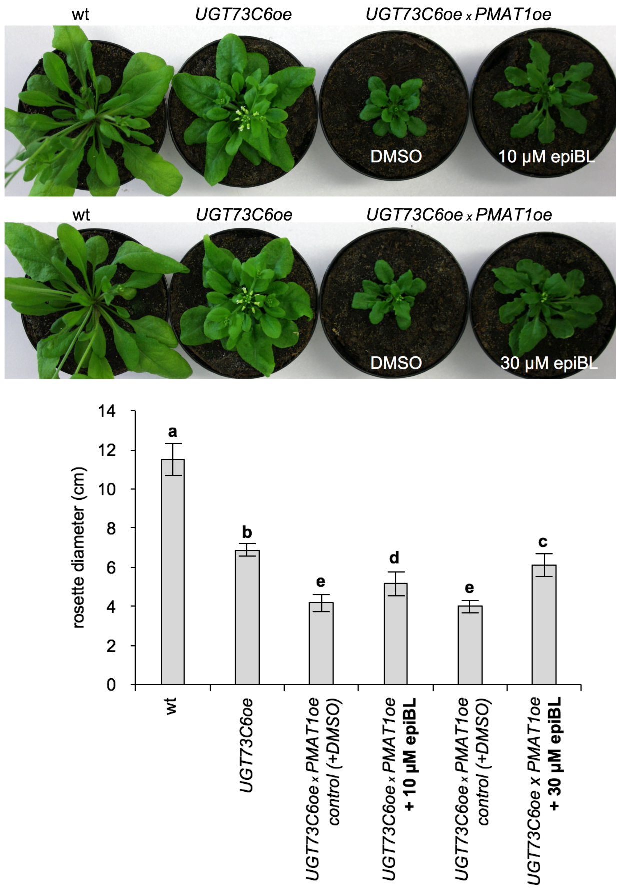

Figure S10. epiBL application rescues aspects of the phenotypes of *UGT73C6oexPMAT1oe* plants.

The plants were grown in LDs at 80 µmol·m^{-2}·s^{-1} of white light and 21°C and sprayed with 10 µM of epiBL, 30 µM of epiBL or DMSO as a control thrice weekly. The treatment was performed for 4 weeks and then the rosette diameters were measured. Top: photos of representative plants. Bottom: mean and SD of the results of 10 measured plants. Letters show significant differences ($p < 0.05$, one-way ANOVA, Tukey post-hoc test).

```
TATAGAATTCATTTAGGTGACACTATAGAAGGGTAATACGACTCACTATAGCAGTGAAGATTGACCATCTC
A
    EcoRI SP6 RNA polymerase promoter
                                 T7 RNA polymerase promoter

CAAAAGCTGTTACGTGCTTGTAACACACTACACACTCGTTTTGTATTCGAGAAGTAGTTGCAACAACGGTC
C
                  barley yellow dwarf virus 5' UTR

CCTTATTGCCTGACAAGCTGAGGGCCACCCTTCTATCCCCACCGCGCGATCGCCATGGAGATCTCGCGGCC
G
                                                      START codon
                                                     NcoI  BglII  NotI

CGAGCAAAAATTAATCAGCGAAGAGGATTTATAACTCGAGAGTGAAGACAACACCACTAGCACAAATCGGA
T
 Myc-tag                             STOP codon

CCTGGGAAACAGGCAGAACTTCGGTTCATAAGCTCGGGTAGGCTGTCAACCTACCGCCGTATCGTATTGTG
T
          barley yellow dwarf virus 3' translation enhancer

TTGTCTAGATACGTATCGCGAGTCGACCTGCAGGCATGCAAGCTGATCCGGCTGCTAACAAAGCCCGAAAG
G

AAGCTGAGTTGGCTGCTGCCACCGCTGAGCAATAACTAGCATAACCCCTTGGGGCCTCTAAACGGGTCTTG
A
                                    T7 terminator

GGGGTTTTTTGAAGCTTTATA
           HindIII
```

Figure S11. Sequence of the synthetic DNA used for cloning of the malonyltransferases into pUC SP6.
